# M/BiOCl‐(M = Pt, Pd, and Au) Boosted Selective Photocatalytic CO_2_ Reduction to C_2_ Hydrocarbons via *CHO Intermediate Manipulation

**DOI:** 10.1002/advs.202400934

**Published:** 2024-07-18

**Authors:** Qiong Liu, Chengbo Bai, Chengxin Zhu, Wenjin Guo, Guangfang Li, Sheng Guo, Devesh Kripalani, Kun Zhou, Rong Chen

**Affiliations:** ^1^ State Key Laboratory of New Textile Materials and Advanced Processing Technologies Wuhan Textile University Wuhan 430200 P. R. China; ^2^ School of Chemistry and Environmental Engineering Wuhan Institute of Technology Donghu New & High Technology Development Zone Wuhan 430205 P. R. China; ^3^ Key Laboratory of Material Chemistry for Energy Conversion and Storage (Ministry of Education) Hubei Key Laboratory of Material Chemistry and Service Failure Huazhong University of Science and Technology Wuhan 430074 P. R. China; ^4^ School of Mechanical and Aerospace Engineering Nanyang Technological University 50 Nanyang Avenue Singapore 639798 Singapore; ^5^ Nanyang Environment and Water Research Institute Nanyang Technological University 1 CleanTech Loop Singapore 637141 Singapore

**Keywords:** *CHO intermediate, *CO adsorption, H_2_O activation, OC─CHO coupling, photocatalytic CO_2_ reduction

## Abstract

Selective CO_2_ photoreduction to C_2_ hydrocarbons is significant but limited by the inadequate adsorption strength of the reaction intermediates and low efficiency of proton transfer. Herein, an ameliorative *CO adsorption and H_2_O activation strategy is realized via decorating bismuth oxychloride (BiOCl) nanostructures with different metal (Pt, Pd, and Au) species. Experimental and theoretical calculation results reveal that distinct *CO binding energies and *H acquisition abilities of the metal cocatalysts mediate the CO_2_ reduction activity and hydrocarbon selectivity. The relatively moderate *CO adsorption and *H supply over Pd/BiOCl endows it with the lowest free energy to generate *CHO, leading to its highest activity of hydrocarbon production. Specifically, the Pt cocatalyst can efficiently participate in H_2_O dissociation to deliver more *H for facilitating the protonation of the *CHO and *CHOH, thereby favoring CH_4_ production with 76.51% selectivity. A lower *H supply over Pd/BiOCl and Au/BiOCl results in a large energy barrier for *CHO or *CHOH protonation and thus a more thermodynamically favored OC─CHO coupling pathway, which endows them with vastly increased C_2_ hydrocarbon selectivity of 81.21% and 92.81%, respectively. The understanding of efficient C_2_ hydrocarbon production in this study sheds light on how materials can be engineered for photocatalytic CO_2_ reduction.

## Graphical abstract

An ameliorative *CO adsorption and H_2_O activation strategy is realized via decorating different cocatalytic metal (Pt, Pd, and Au) on bismuth oxychloride (BiOCl) nanostructures, which result in selective CO_2_ photoreduction to C_2_ hydrocarbons via *CHO intermediate manipulation.

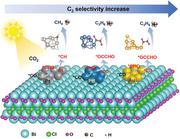

## 1 Introduction

Photocatalytic CO_2_ reduction, which uses light sources as the sole energy input to drive CO_2_ reduction to chemical fuels, is attractive as a means of reducing atmospheric CO_2_ levels while alleviating our dependence on fossil fuels.^[^ ^1^ ^]^ Nevertheless, the photocatalytic CO_2_ reduction reaction (PCRR) is an extremely complex process involving multi‐step proton‐coupled electron transfer (PCET) reactions, resulting in a wide distribution of products and low selectivity of high‐value target products.^[^ ^2^ ^]^ In comparison to mono‐carbon (C_1_) products (i.e., CO and CH_4_), multicarbon (C_2+_) hydrocarbon products, with a higher energy density, larger chemical value, and broader application range, are more valuable.^[^ ^3^ ^]^ In particular, C_2_ products are difficult to obtain in PCRR due to the competition with H─H and C─H bond formation and the kinetic barrier of C─C coupling.^[^ ^4^ ^]^ At the same time, the lack of in‐depth understanding of the catalytic mechanism exacerbates the difficulty in advancing the catalyst design to promote the efficiency of C_2_ conversion.^[^ ^4b^ ^]^ Therefore, the development of efficient photocatalysts for the reduction of CO_2_ to C_2_ hydrocarbons and the exploration of the mechanism is a highly challenging and significant task.

In the reaction pathway from CO_2_ to C_2_ hydrocarbons, the main limitation is the challenging C─C coupling process.^[^ ^5^ ^]^ Recently, theoretical calculations and experimental investigations have proposed that the formation of C─C bonds via an OC─CHO coupling pathway is thermodynamically and kinetically more favorable than the dimerization of *CO into *OCCO.^[^ ^4^, ^5^ ^]^ A moderate adsorption strength of the catalyst for *CO is the key to ensure its further hydrogenation to *CHO, which is essential for the *OC─CHO pathway.^[^ ^6^ ^]^ Meanwhile, the role of *H cannot be ignored.^[^ ^3^, ^7^ ^]^ The hydrogenation process plays a significant role as a rate‐determining step in PCRR, highlighting the importance of having an efficient supply of *H species. Specifically, stable water undergoes splitting to generate absorbed *H (H_2_O+*+e^−^→*H+OH^−^) as the initial step in this process.^[^ ^7^, ^8^ ^]^ Subsequently, *H actively participates in the hydrogenation reaction with adsorbed *CO_2_ to form the key intermediate *CHO.^[^ ^9^ ^]^ *CHO can also continuously combine with *H to form CH_4_ (*CHO+*H→CH_4_), which serves as a competing reaction in the production of C_2_ hydrocarbons (*CHO+*CO→C_2_).^[^ ^7b^ ^]^ In light of this, manipulating the adsorption and coverage of *CO and *H on the catalyst surface is crucial for regulating the formation of the *OC─CHO dimer species while suppressing the further hydrogenation of *CHO to generate CH_4_.

The metal‐coated catalysts strategy is a promising approach to tune the electronic structure of catalysts to optimize intermediate adsorption for performance improvement.^[^ ^10^ ^]^ For example, the Au─CeO_2_ microinterface facilitates C_2_H_6_ production by promoting C─C coupling by clustering a large number of electrons around the C atoms.^[^ ^10b^ ^]^ After replacing Au with Cu and Pt, only CH_4_ and CO are produced, indicating that the type of supporting metal also considerably affects the coupling of the C─C bond. However, few studies have correlated the product selectivity of catalytic materials with the intrinsic properties of the cocatalytic metals. In fact, different transition metals have distinct *H and *CO adsorption energies and H_2_O dissociation abilities, which are closely related to the corresponding electronic structure of the metal surface.^[^ ^11^ ^]^ For example, Au and Ag can weakly adsorb *CO and *H,^[^ ^6^, ^11^ ^]^ whereas Pt and Pd have under potential deposited hydrogen and strong *CO adsorption strength.^[^ ^2^, ^11^ ^]^ Inspired by the above analysis, constructing different cocatalytic transition metals on a catalyst surface to tune its *CO adsorption and *H supply can be expected to achieve selective regulation of C_1_ and C_2_ hydrocarbons in PCRR.^[^ ^7^, ^9^ ^]^ It is worth to systematically explore the regulation rules of surface *CO and *H adsorption and coverage of different cocatalytic transition metals and its effects on the selectivity of C_1_ and C_2_ hydrocarbons in PCRR, which will provide essential theoretical guidance for the design of C_2_ hydrocarbon‐producing catalysts.

BiOCl, with its distinctive layered structure and electronic and optical properties, is widely used in the field of photocatalysis.^[^ ^12^ ^]^ Although it was found to exhibit activity in PCRR, the main product is also CO.^[^ ^12a,b^ ^]^ Herein, a strategy to regulate *CO adsorption and *H supply by transition metal cocatalysis was proposed to improve the PCRR activity and C_2_ hydrocarbon selectivity of BiOCl. M/BiOCl (M = Pt, Pd, and Au) nanostructures were employed as model catalysts. In this study, we selected Au, Pt, and Pd as representative elements due to their distinct *H and *CO adsorption energies and H_2_O dissociation abilities. Computationally, the theoretical feasibility of this strategy was first investigated by calculating the *CO adsorption energy and free energy of H_2_O dissociation on BiOCl and M/BiOCl. Experimentally, M/BiOCl was prepared by deposition–precipitation and NaBH_4_ reduction. The production of hydrocarbons in PCRR is largely facilitated by M/BiOCl; in particular, Au/BiOCl and Pd/BiOCl significantly increase the activity and selectivity of CO_2_ conversion to C_2_H_6_. Density functional theory (DFT) calculations and in situ diffuse reflectance infrared Fourier transform spectroscopy (DRIFTS) were also performed to gain insight into the mechanism behind the different activity and selectivity of BiOCl and M/BiOCl. Subsequently, typical cocatalytic metal (Pt, Pd, and Au) species were also supported on additional substrates (P25 and g‐C_3_N_4_), and the common characteristics of the cocatalytic metal and individual characteristics of the substrates in regulating the C_1_ and C_2_ hydrocarbon selectivity of PCRR were discussed. The results of this work provide a new perspective and theoretical basis for the design of C_2_‐producing catalysts, facilitating the development of universal catalyst design strategies.

## 2 Results and Discussion

**Figure 1:**
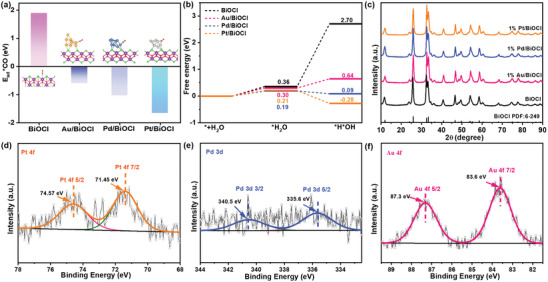
Calculated a) *CO adsorption energies and b) free energy of H_2_O dissociation on BiOCl and M/BiOCl (M = Pt, Pd, and Au). c) XRD patterns of BiOCl and 1% M/BiOCl (M = Pt, Pd, and Au). XPS high‐resolution spectrum of d) Pt 4f of 1% Pt/BiOCl, e) Pd 3d of 1% Pd/BiOCl, and f) Au 4f of 1% Au/BiOCl.

To exploit the metal‐mediated regulation of surface *CO and *H adsorption and coverage, density functional theory (DFT) calculations were performed based on the computational models of BiOCl‐(001), Pt/BiOCl‐(001), Pd/BiOCl‐(001), and Au/BiOCl‐(001) (Figure S1, Supporting Information). As displayed in **Figure** **1**a, the decreased adsorption energies (*E*_ad_*CO) of M/BiOCl (M = Pt, Pd, and Au) than that of BiOCl demonstrated the improved *CO adsorption strength. In particular, Pt/BiOCl exhibits lower *E*_ad_*CO (−1.66 eV) than Pd/BiOCl (−1.04 eV) and Au/BiOCl (−0.60 eV), suggesting that the CO adsorption strength of M/BiOCl is closely related to that of the cocatalytic metal (the CO adsorption strength is in the order of Pt>Pd>Au^[^ ^11^ ^]^). In addition, after the introduction of Au, Pd, and Pt on BiOCl, the free energy of H_2_O dissociation dropped from 2.34 to 0.34, −0.10, and −0.49 eV, respectively (Figures 1b and S2, Supporting Information), confirming that the supported metal accelerates H_2_O dissociation and facilitates proton‐feeding.^[^ ^9^, ^13^ ^]^ The *H acquisition ability of Pt/BiOCl is the best, followed by Pd/BiOCl, and then Au/BiOCl.

Experimentally, BiOCl with exposed facets of (001) was prepared via a simple deposition–precipitation process. The M/BiOCl (M = Pt, Pd, and Au) photocatalysts were prepared using a wet‐chemical approach with sodium borohydride as the reducing agent. As illustrated in Figure 1c, all characteristic diffraction peaks of BiOCl and 1% M/BiOCl (M = Pt, Pd, and Au) were well‐indexed to the standard X‐ray diffraction (XRD) pattern of BiOCl (PDF 6‐249), demonstrating that metal loading did not affect the crystallinity and fine structure of BiOCl.^[^ ^14^ ^]^ No diffraction peaks of Au, Pt, and Pd were observed, which might be attributed to the metal size, crystallinity, and content being beyond the XRD detection limit. Figure 1d shows that the Pt 4f 7/2 peak at 71.5 eV and Pt 4f 5/2 peak at 74.6 eV can be attributed to metallic Pt^0^.^[^ ^15^ ^]^ The binding energy at 340.5 and 335.6 eV, corresponding to the Pd 3d 3/2 and Pd 3d 5/2 peaks, were assigned to metallic Pd^0^ (Figure 1e).^[^ ^16^ ^]^ The observed Au 4f 7/2 and Au 4f 5/2 peaks located at 83.6 and 87.3 eV, respectively, agree well with the values of metallic Au^0^ (Figure 1f).^[^ ^17^ ^]^

**Figure 2:**
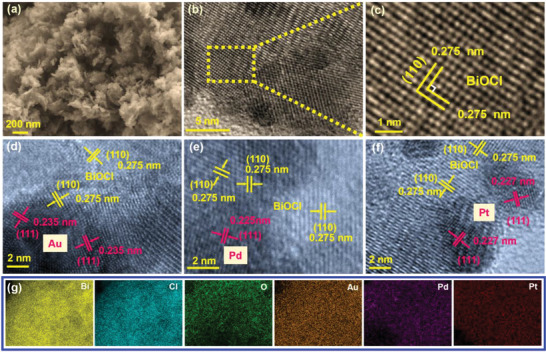
a) SEM image of 1% Au/BiOCl, HRTEM images of b,c) BiOCl, d) 1% Au/BiOCl, e) 1% Pd/BiOCl and f) 1% Pt/BiOCl, and g) EDX elemental mapping of 1% M/BiOCl (M = Au, Pd, and Pt).

According to the scanning electron microscope (SEM) images obtained (Figure S3a, Supporting Information), BiOCl displayed an irregular nanosheet structure with disordered arrangement. 1% M/BiOCl (M = Au, Pt, and Pd) have a similar morphology as that of BiOCl (**Figures** **2**a and S3, Supporting Information), albeit with slightly different Brunauer‐Emmett‐Teller (BET) specific surface areas (Figure S4, Supporting Information). High‐resolution transmission electron microscope (HRTEM) imaging showed clear lattices with an interplanar spacing of 0.275 nm, corresponding to the (110) atomic planes of BiOCl (Figure 2b,c). It also showed clearer lattice stripes for Au in 1% Au/BiOCl with a lattice spacing of 0.235 nm, which was indexed to the (111) facets of metallic Au (Figure 2d). The distinguishable facet distance of 0.225 and 0.227 nm displayed in the HRTEM images of 1% Pd/BiOCl (Figure 2e) and 1% Pt/BiOCl (Figure 2f) originated from the (111) plane of Pd and Pt, respectively. Energy dispersive X‐ray spectroscopy (EDX) evidenced the uniform distribution of Bi, Cl, O, and metal element (Au, Pt, and Pd) throughout M/BiOCl (Figure 2g). The weight percentage of Pt, Pd, and Au was measured to be around 1.04, 1.18, and 1.07 wt%, respectively (Figure S5, Supporting Information).

**Figure 3:**
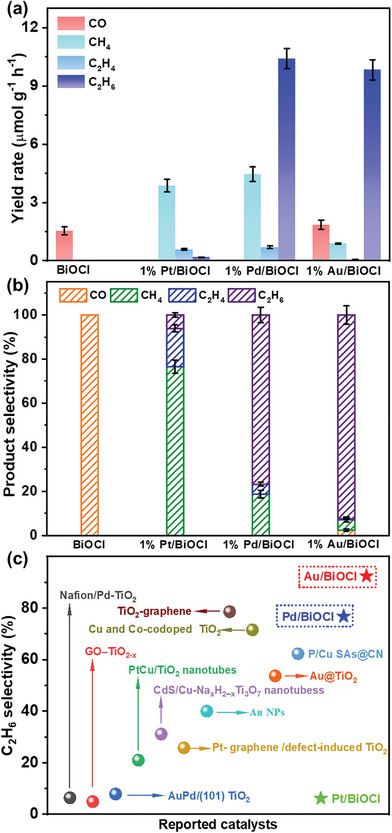
a) The yield rate of BiOCl and 1% M/BiOCl (M = Pt, Pd, and Au) samples after 4 h of PCRR, b) the product selectivity of BiOCl and M/BiOCl, and c) the selectivity of C_2_H_6_ of 1% M/BiOCl in comparison with recent reports.

Then, we evaluate the PCRR performance of the as‐prepared material. According to gas chromatography (GC) analysis (Figure S6a, Supporting Information), only a slight amount of CO was generated over pure BiOCl, without any indication of additional reductive carbon‐containing products. However, CH_4_, C_2_H_6_, and C_2_H_4_ products were detected in the PCRR system of 1% M/BiOCl (M = Pt, Pd, and Au) (Figure S6b–d, Supporting Information). No byproduct of H_2_ is generated in all systems. Although the photocatalytic reduction of CO_2_ to carbonaceous products using H_2_O as the oxidant assumes the formation of O_2_, no O_2_ was detected in the PCRR system of M/BiOCl (M = Pt, Pd, and Au) samples. This could potentially be due to the consumption of detectable O_2_ in replenishing oxygen vacancies on the M/BiOCl catalyst surface during the photocatalytic process, thereby resulting in its absence during the CO_2_ reduction reaction.^[^ ^18^ ^]^ Compared to BiOCl, M/BiOCl exhibited significantly improved photocatalytic activity, especially in the production of hydrocarbon products (**Figure** **3**a). As illustrated in Figure S7 (Supporting Information), BiOCl samples supported with 1% metal (Pt, Pd, and Au) demonstrated enhanced PCRR activity relative to those supported with 0.5% and 1.5% metal (Pt, Pd, and Au). Specifically, 1% Pd/BiOCl exhibited the best PCRR activity, whereby the yield rate of CH_4_, C_2_H_4_, and C_2_H_6_ was 4.45, 0.70, and 10.40 µmol g^−1^ h^−1^, respectively (Figure 3a). In accordance with literature,^[^ ^19^ ^]^ the solar‐to‐CO/CH_4_/C_2_H_4_/C_2_H_6_ conversion efficiency (*η*) of BiOCl, 1% Pt/BiOCl, 1% Pd/BiOCl, and 1% Au/BiOCl were calculated to be approximately 0.0006%, 0.0063%, 0.0299%, and 0.0237%, respectively. Notably, BiOCl and M/BiOCl (M = Au, Pt, and Pd) exhibited different product selectivity during PCRR. As shown in Figure 3b and Table S1 (Supporting Information), BiOCl exhibited 100% selectivity of CO, while it was only 2.49% for 1% Au/BiOCl, and even drastically reduced to 0% for 1% Pt/BiOCl and 1% Pd/BiOCl. It was noteworthy that the selectivity of CH_4_ over 1% Pt/BiOCl (76.51%), 1% Pd/BiOCl (18.79%), and 1% Au/BiOCl (4.70%) was gradually decreased, while the selectivity of C_2_ products presented an opposite trend, which accounted for 23.49%, 81.21%, and 92.81%, respectively. Impressively, the C_2_H_6_ selectivity of 1% Au/BiOCl (92.46%) was much higher than that of other photocatalysts reported in literature (Figure 3c and Table S2, Supporting Information).

In order to investigate whether the use of mannitol solvent in BiOCl preparation could introduce carbon contaminants, we synthesized BiOCl‐1 samples using deionized water instead of mannitol solution under identical conditions (Figure S8, XRD, Supporting Information). Although the PCRR activity of the 1% Pd/BiOCl‐1 sample was observed to be lower than that of the 1% Pd/BiOCl sample (Figure S9, Supporting Information), this finding confirms that the formation of CH_4_, C_2_H_4_, and C_2_H_6_ products is not attributed to mannitol. The difference in their PCRR activity could potentially arise from variations in the physical properties such as morphology and specific surface area (Figures S10 and S11, SEM and BET, Supporting Information). Control experiments (Figure S12, Supporting Information) indicated that there was no product formation under dark conditions or in the absence of a catalyst, confirming that the reduction of CO_2_ over M/BiOCl was a characteristic photocatalytic process. Once CO_2_ is replaced by Ar, only a few products are detected, suggesting that any other possible interference factors are negligible. Additionally, an isotope‐labeled ^13^CO_2_ experiment was performed using the same conditions with a 1% Pd/BiOCl sample, which revealed the presence of signals corresponding to ^13^CH_4_ (m/z = 17) and ^13^C_2_H_6_ (m/z = 32) (Figure S13, Supporting Information). This further substantiates the capability of 1% Pd/BiOCl in catalyzing CO_2_ reduction under light irradiation.

**Figure 4:**
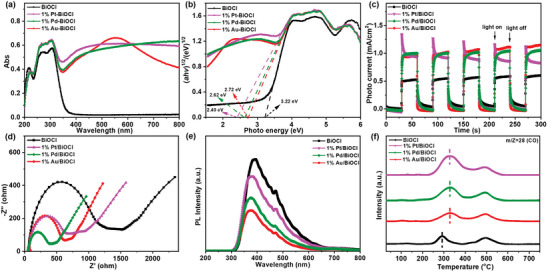
The a) DRS, b) bandgaps, c) transient photocurrent responses under UV–visible light irradiation, d) electrochemical impedance spectroscopy (EIS) Nyquist plots, e) PL spectra, and f) CO‐TPD of BiOCl and 1% M/BiOCl (M = Pt, Pd, and Au) samples.

Additionally, the UV–vis diffuse reflectance spectra (DRS), photochemical properties, and temperature‐programmed desorption (TPD) were analyzed to investigate the interaction between the co‐catalytic metal and BiOCl. As shown in **Figures** **4**a and S14 (Supporting Information), the introduction of Au resulted in additional light harvesting in the visible region with a peak maximum at 557 nm, which was ascribed to the localized surface plasmon resonance (LSPR) absorption band of Au nanoparticles.^[^ ^20^ ^]^ As for the LSPR effect of Pt and Pd, the 1% Pt/BiOCl and 1% Pd/BiOCl samples show a clear enhanced absorption at 400–800 nm.^[^ ^21^ ^]^ The direct bandgap energies (*E*_g_) of 1% M/BiOCl (M = Pt, Pd, and Au) were correspondingly reduced (Figure 4b). Significantly enhanced photocurrent intensity, smaller charge transfer resistance, and declined photoluminescence (PL) intensity were observed over the M/BiOCl samples (Figure 4c–e), confirming the positive role of Au, Pt, and Pd nanoparticles in charge generation, transformation, and separation.^[^ ^22^ ^]^ Furthermore, according to the CO‐TPD‐mass spectra in Figure 4f, the CO desorption temperature of M/BiOCl shifted to higher temperatures with an increased peak desorption area than that of BiOCl, revealing that the co‐catalytic metal improved CO adsorption in terms of both capacity and strength, which is in good agreement with the theoretical calculation (Figure 1a).^[^ ^10a^ ^]^ The enhanced photoelectric and adsorption properties not only improve the PCRR activity, but also facilitate the formation of C_1_ and C_2_ hydrocarbon products by chemical reactions involving multiple e^−^/H^+^ transfer processes.^[^ ^20^, ^21^ ^]^

**Figure 5:**
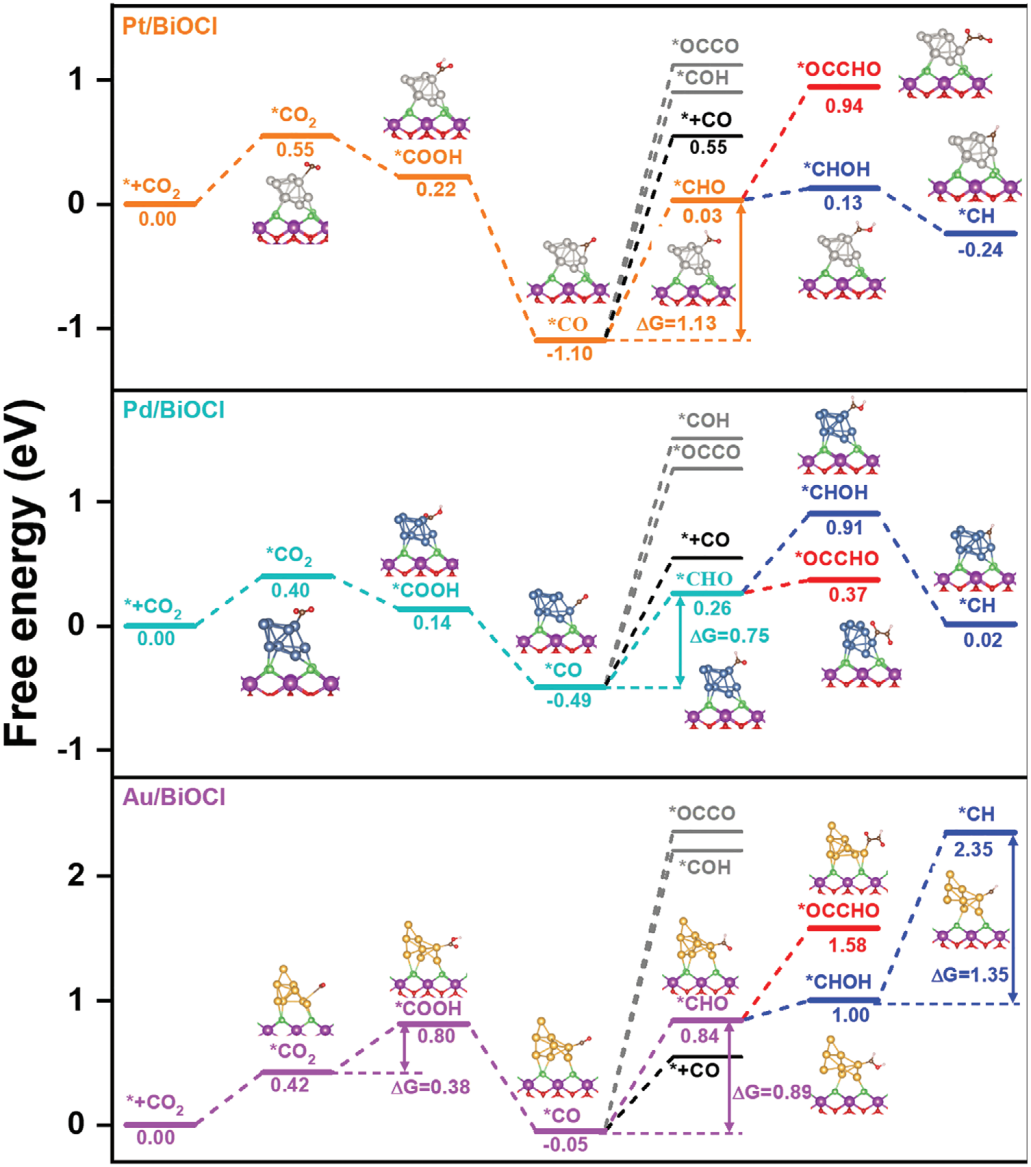
Calculated free energy of CO_2_ reduction on BiOCl and M/BiOCl (M = Pt, Pd, and Au) for the photoreduction of CO_2_ to CH_4_ and C_2_ hydrocarbons with H_2_O.

Gibbs free energy calculations were then performed to further understand the specific structure and CO_2_ reduction mechanism of the constructed M/BiOCl (M = Pt, Pd, and Au) photocatalyst (**Figures** **5** and S15–S19, Supporting Information). As displayed in Figure S15 (Supporting Information), in the process of CO_2_ reduction to CO over BiOCl, the formation of *COOH was the rate‐limiting step because it had the highest energy barrier (Δ*G* = 2.2 eV). After the introduction of Pt, Pd, and Au, the calculated free energy of *CO_2_ hydrogenation to *COOH significantly decreased to −0.33, −0.26, and 0.38 eV, respectively (Figure 5), which might be attributed to the improved proton‐feeding rate of M/BiOCl that was beneficial for the protonation of *CO_2_ to form *COOH.^[^ ^9^ ^]^ Thus, the PCRR activity of M/BiOCl is enhanced compared to that of BiOCl. Furthermore, the free energy of *CO desorption to CO gas (−1.89 eV) is much lower than that of any of the other reaction pathways (Figure S15, Supporting Information), demonstrating that CO can readily escape from the surface as the final and selective product on BiOCl. Nevertheless, enhanced CO adsorption on M/BiOCl (M = Au, Pd and Pt) leads to increased free energies of *CO desorption (0.60, 1.04, and 1.65 eV, respectively) (Figure 5), effectively demonstrating the decreased selectivity of CO over M/BiOCl, which is in good agreement with the experimental results. The stabilized *CO on M/BiOCl will undergo further hydrogenation to form *CHO rather than *COH, and will also not directly dimerize to give *OCCO because of their higher uphill energy.^[^ ^23^ ^]^ The free energy of *CHO formation on Pd/BiOCl (0.75 eV) was lower than that of Au/BiOCl (0.89 eV) and Pt/BiOCl (1.13 eV), which might be attributed to the lower *H coverage on Au/BiOCl and excessive binding of *CO on Pt/BiOCl, which inhibited the hydrogenation of *CO. Pd/BiOCl displayed the best activity toward CO_2_ reduction to hydrocarbons, indicating that the hydrogenation of *CO to form *CHO was the key step in the formation of hydrocarbons.

The *CO adsorption strength and *H coverage on M/BiOCl greatly affect the reaction pathway of *CHO, and thereby the selective formation of CH_4_ (*CHO+*H→CH_4_) and C_2_ hydrocarbons (*CHO+*CO→C_2_).^[^ ^7b^ ^]^ As for Pt/BiOCl, Pt co‐catalytic sites can efficiently participate in H_2_O dissociation to deliver more *H for facilitating the continuous protonation of *CHO (Δ*G* = 0.10 and −0.37 eV), rather than *CHO coupling with *CO to form *OC─CHO (Δ*G* = 0.91 eV). Since *OC─CHO has a higher free energy (Figure 5), CH_4_ is highly favored as the generated product. In contrast, the slightly reduced H_2_O dissociation activity of Pd/BiOCl and Au/BiOCl results in a lower *H supply and a large energy barrier of *CHO or *CHOH protonation. It was also found that the free energy of *OC─CHO formation on Pd/BiOCl (Δ*G* = 0.11 eV) was much lower than that on Au/BiOCl (Δ*G* = 0.74 eV) and Pt/BiOCl (Δ*G* = 0.91 eV) (Figure 5), suggesting that the relatively moderate binding of *CO on Pd/BiOCl was beneficial for the coupling of *CHO and *CO into *OC─CHO. As for Pd/BiOCl, the formation of *OCCHO intermediates is more thermodynamically favored than *CHO undergoing hydrogenation to form *CHOH (Δ*G* = 0.11 vs 0.65 eV), thereby its C_2_ hydrocarbon selectivity was much higher than that of CH_4_.^[^ ^4b^ ^]^ Meanwhile, the further hydrogenation of *CHOH to form *CH on Au/BiOCl needs a very high uphill energy of 1.35 eV, which greatly inhibits the formation of CH_4_ and endows Au/BiOCl with the highest C_2_ hydrocarbon selectivity.

**Figure 6:**
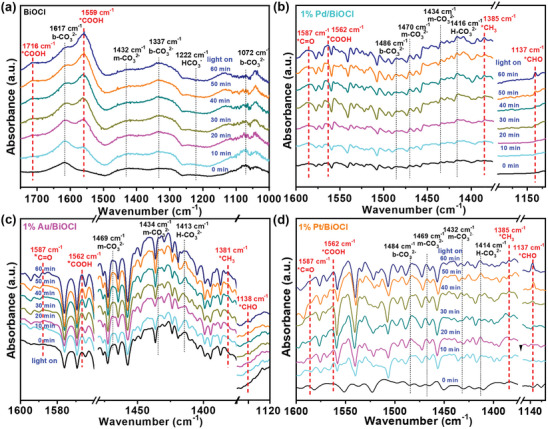
In situ DRIFTS of CO_2_ reduction over a) BiOCl, b) 1% Pd/BiOCl, c) 1% Au/BiOCl, and d) 1% Pt/BiOCl at different illumination times (CO_2_ adsorption for 30 min in the dark, and under illumination for 10, 20, 30, 40, 50, and 60 min).

In situ DRIFTS detection of the CO_2_ reduction intermediates was used to provide direct evidence for the identification of the reaction pathway. As shown in **Figure** **6**a, the initial reactive adsorption of CO_2_ on BiOCl leads to the formation of carbonate (CO_3_^2−^) and bicarbonate (HCO_3_^−^) species.^[^ ^24^ ^]^ After illumination, the peaks at 1617 cm^−1^ and 1072 cm^−1^ gradually weakened. Importantly, new peaks at 1559 and 1716 cm^−1^ corresponding to the stretching of *COOH were gradually strengthened with the increase of irradiation time, which was regarded as the key intermediate during the reduction of CO_2_ to CO.^[^ ^25^ ^]^ As shown in Figure 6b, exposure of 1% Pd/BiOCl to CO_2_ also leads to the formation of surface carbonates, but with an absorption pattern that was somewhat distinct from that seen in the case of BiOCl.^[^ ^24^, ^26^ ^]^ After illumination, the peaks attributed to *COOH (1562 cm^−1^) were also recorded. Differently, the characteristic peaks of *CHO at 1137 cm^−1^ and *CH_3_ at 1385 cm^−1^ were observed and strengthened with illumination time, which are crucial intermediates for CO_2_ reduction to CH_4_.^[^ ^10^, ^27^ ^]^ As shown in Figure 6c,d, similar surface adsorbed species and adsorbed reaction intermediates were also found for 1% Au/BiOCl and 1% Pt/BiOCl. Specially, a relatively obvious peak related to C═O stretching of the *OCCHO intermediate at 1587 cm^−1^ appeared for 1% Pd/BiOCl (Figure 6b) and 1% Au/BiOCl (Figure 6c), which provides strong experimental evidence for C─C coupling during photocatalytic CO_2_ reduction.^[^ ^4^, ^27^, ^28^ ^]^

**Figure 7:**
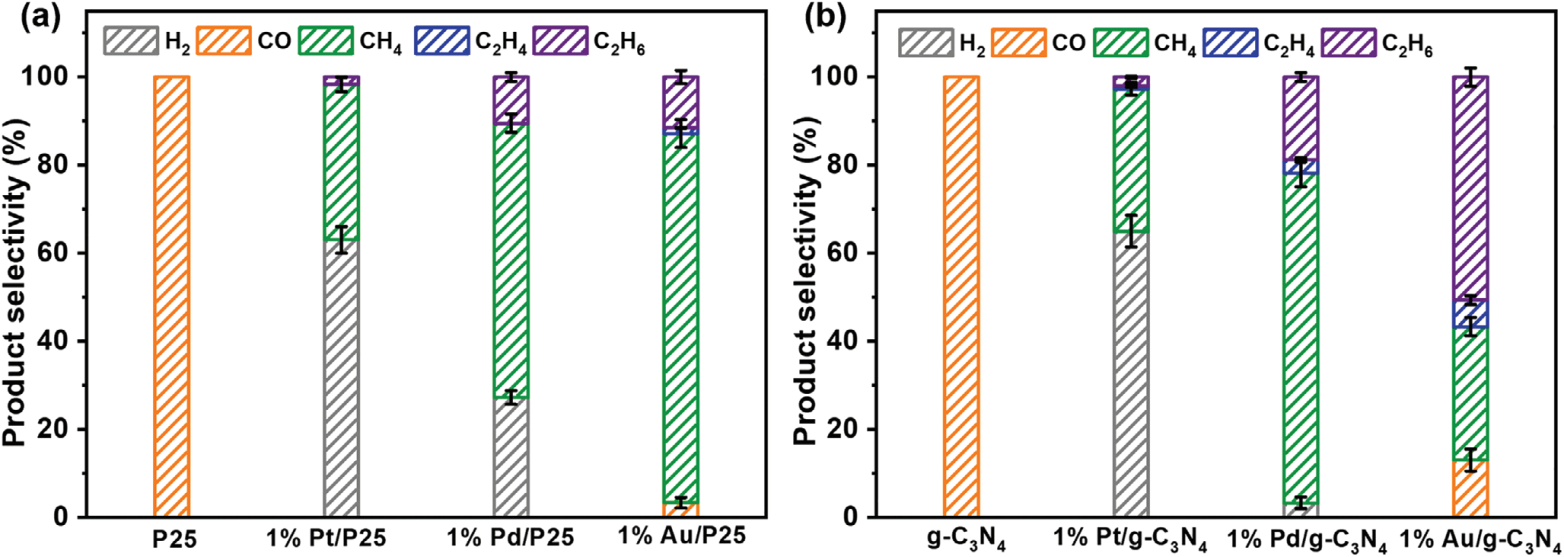
The selectivity of different products over a) P25 and 1% Pt‐, 1% Pd‐, and 1% Au‐supported P25, and b) over g‐C_3_N_4_ and 1% Pt‐, 1% Pd‐, and 1% Au‐supported g‐C_3_N_4_ samples after 4 h of PCRR.

Meanwhile, this strategy is universal for other common semiconductor catalysts (P25 and g‐C_3_N_4_, Figure S20, XRD and DRS results, Supporting Information). As displayed in Table S1 and Figure S21 (Supporting Information), and **Figure** **7**, Pt, Pd, and Au all present synergistic effects with P25 and g‐C_3_N_4_ to improve hydrocarbon production activity and tune its selectivity. First, the stronger the *CO adsorption of the cocatalytic metal, the lower the selectivity of CO of the corresponding metal‐supported catalyst. Second, Au and Pd cocatalysts are more favorable than Pt for forming C_2_ products. Nevertheless, there were a lot of H_2_ by‐products being produced over 1% M/P25 and 1% M/g‐C_3_N_4_ (M = Pt and Pd). Interestingly, the H_2_ selectivity of 1% Pt/P25, 1% Pd/P25, and 1% Au/P25 gradually decreases, while the CH_4_ and C_2_ hydrocarbon selectivity gradually increases. 1% M/g‐C_3_N_4_ (M = Pt, Pd, and Au) also exhibited a similar trend, except for 1% Au/g‐C_3_N_4_ which presented decreased CH_4_ selectivity and increased C_2_H_6_ selectivity. Therefore, it can be concluded that Pt has under potential deposited hydrogen and is more favorable for generating products with a relatively elevated hydrogen content than Pd or Au on the same substrate. In contrast, the selectivity of products with a relatively low hydrogen content increases in PCRR. Evidently, the yield and selectivity of C_2_H_6_ over M/BiOCl (M = Pt, Pd, and Au) were much higher than that of identical metal‐supported P25 and g‐C_3_N_4_, suggesting the critical role of an active substrate for the improvement of PCRR activity and selectivity of desirable products.

## 3 Conclusions

In summary, M/BiOCl (M = Au, Pd, and Pt) achieved photocatalytic conversion of CO_2_ to hydrocarbon products, which was attributed to their improved photoelectric performance, CO adsorption, and H_2_O dissociation activity. Specially, the relatively modest binding of *CO and H_2_O dissociation activity of Pd/BiOCl results in the lowest energy barrier for *CHO formation, and thus its highly efficient PCRR toward hydrocarbons as compared to Au/BiOCl and Pt/BiOCl. More importantly, distinct *CO binding energies and *H acquisition abilities of the different co‐catalytic metals achieved the regulation of C_1_ and C_2_ product selectivity. The Pt cocatalyst exhibits the best H_2_O dissociation activity and is more favorable for facilitating the protonation of *CHO and *CHOH, and thereby the formation of products with a relatively high hydrogen content (CH_4_). In contrast, the H_2_O dissociation activity of the Pd and Au cocatalysts decreased gradually, and the OC─CHO coupling pathway was thermodynamically favored rather than *CHO hydrogenation, thus the selectivity of products with a relatively low hydrogen content (C_2_ hydrocarbons) was gradually improved. Similar tuning effects were also found over M/P25 and M/g‐C_3_N_4_ (M = Pt, Pd, and Au). It is essential to note that when keeping the active metal unchanged, the choice of substrate would play a crucial role in product selectivity. BiOCl is a good candidate substrate for the selective production of C_2_H_6_. On the other hand, the size and crystal face of the loaded metal particles may also have a certain effect on the selectivity of the product, which is worthy of further investigation in future work. Regulating *CO adsorption and *H acquisition is a promising strategy to achieve efficient selectivity during photocatalytic CO_2_ reduction reactions.

## Conflict of Interest

The authors declare no conflict of interest.

## Supporting information

Supporting Information

## Data Availability

The data that support the findings of this study are available from the corresponding author upon reasonable request.
