# Supplementary material for: M/BiOCl‐(M = Pt, Pd, and Au) Boosted Selective Photocatalytic CO2 Reduction to C2 Hydrocarbons via *CHO Intermediate Manipulation
Source: Adv Sci (Weinh). 2024 Jul 18;11(35):2400934. doi: 10.1002/advs.202400934 (PMC11425252; doi:10.1002/advs.202400934)
Supplement: Supplementary file 1 — Supporting Information [file ADVS-11-2400934-s001.docx]

Supporting Information

M/BiOCl-(M=Pt, Pd, and Au) Boosted Selective Photocatalytic CO_2_ Reduction to C_2_ Hydrocarbons *via* *CHO Intermediate Manipulation

*Qiong Liu^1,2^, Chengbo Bai^1^, Chengxin Zhu^1^, Wenjin Guo^1^, Guangfang Li^3^, Sheng Guo^1^,*

*Devesh Kripalani^4^, Kun Zhou^4,5,^**, *Rong Chen^1,^**

*^1^ State Key Laboratory of New Textile Materials and Advanced Processing Technologies, Wuhan Textile University, Wuhan 430200, PR China*

*^2^ School of Chemistry and Environmental Engineering, Wuhan Institute of Technology, Donghu New & High Technology Development Zone, Wuhan 430205, PR China*

*^3^ Key Laboratory of Material Chemistry for Energy Conversion and Storage (Ministry of Education), Hubei Key Laboratory of Material Chemistry and Service Failure, Huazhong University of Science and Technology, Wuhan 430074, PR China*

*^4^ School of Mechanical and Aerospace Engineering, Nanyang Technological University, 50 Nanyang Avenue, Singapore 639798, Singapore*

*^5^* *Nanyang Environment and Water Research Institute,* *Nanyang Technological University, 1 CleanTech Loop, Singapore, 637141, Singapore*

*Corresponding author:

Prof. Rong Chen, Wuhan Textile University, Wuhan 430200, PR China; E-mail address: rchenhku@hotmail.com; Tel.: +86 13659815698; Fax: +86 27 59367810

Prof. Kun Zhou, Nanyang Technological University, Singapore 639798, Singapore; E-mail address: kzhou@ntu.edu.sg

**Content**

**I. Supplementary Experimental Details**

**II. Supplementary Figures**

**Figure S1.** Computational models of BiOCl-(001) (a), Pt/BiOCl-(001) (b), Pd/BiOCl-(001) (c), and Au/BiOCl-(001) (d).

**Figure S2.** Adsorption configurations of various intermediates involved in H_2_O dissociation on BiOCl (a), Pt/BiOCl (b), Pd/BiOCl (c), and Au/BiOCl (d).

**Figure S3.** SEM images of BiOCl (a), 1% Pt/BiOCl (b), and 1% Pd/BiOCl (c) samples.

**Figure S4.** N_2_ adsorption–desorption isotherm and BET speciﬁc surface areas of BiOCl (a), and 1% Pt/BiOCl, 1% Pd/BiOCl, and 1% Au/BiOCl (b) samples.

**Figure S5.** Energy-dispersive X-ray spectroscopy of 1% Pt/BiOCl (a), 1% Pd/BiOCl (b), and 1% Au/BiOCl (c) samples.

**Figure S6.** Gas chromatograms of the products generated over BiOCl (a), 1% Pt/BiOCl (b), 1% Pd/BiOCl (c), and 1% Au/BiOCl (d) samples during photocatalytic CO_2_ reduction after 0, 2 and 4 h.

**Figure S7.** The yield of different products versus irradiation time for the photocatalytic CO_2_ reduction reaction over BiOCl (a), and 0.5%, 1%, and 1.5% Pt (b), 0.5%, 1%, and 1.5% Pd (c), and 0.5%, 1%, and 1.5% Au (d) supported BiOCl samples.

**Figure S8.** XRD patterns of the BiOCl-1 (a) and 1% Pd/BiOCl-1 (b) samples.

**Figure S9.** SEM images of BiOCl-1 sample (a) and (b).

**Figure S10.** N_2_ adsorption–desorption isotherms and BET speciﬁc surface areas of BiOCl-1 and 1% Pd/BiOCl-1 samples.

**Figure S11.** The yield of different products versus irradiation time for the photocatalytic CO_2_ reduction reaction over BiOCl-1(a) and 1% Pd/BiOCl-1 (b) samples.

**Figure S12.** PCRR control experiments of 1% M/BiOCl (M=Pt, Pd and Au) samples.

**Figure S13.** Isotope labeled ^13^CO_2_ experiment of 1% Pd/BiOCl.

**Figure S14.** The UV–vis diffuse reflectance spectra (DRS) of 0.5%, 1%, and 1.5% Au/BiOCl (a), 0.5%, 1%, and 1.5% Pd/BiOCl (b), and 0.5%, 1%, and 1.5% Pt/BiOCl (c).

**Figure S15.** Calculated free energy of CO_2_ reduction on BiOCl for the photoreduction of CO_2_ to CO with H_2_O.

**Figure S16.** Adsorption configurations of the various intermediates involved in photocatalytic CO_2_ reduction over the BiOCl sample.

**Figure S17.** Adsorption configurations of the various intermediates involved in photocatalytic CO_2_ reduction over the Pt/BiOCl sample.

**Figure S18.** Adsorption configurations of the various intermediates involved in photocatalytic CO_2_ reduction over the Pd/BiOCl sample.

**Figure S19.** Adsorption configurations of the various intermediates involved in photocatalytic CO_2_ reduction over the Au/BiOCl sample.

**Figure S20.** XRD (a and c) and DRS (b and d) of metal-supported (1% Pt, 1% Pd, and 1% Au) P25 (a and b) and metal-supported (1% Pt, 1% Pd, and 1% Au) g-C_3_N_4_ (c and d) samples.

**Figure S21.** The yield rate of P25 and metal-supported (1% Pt, 1% Pd, and 1% Au) P25 (a), and g-C_3_N_4_ and metal-supported (1% Pt, 1% Pd, and 1% Au) g-C_3_N_4_ (b) samples after 4 h of photocatalytic CO_2_ reduction.

**III. Supplementary Tables**

**Table S1.** The yield and selectivity (%) of different products over 1% Pt, 1% Pd, and 1% Au supported BiOCl, P25 and g-C_3_N_4_ samples after 4 h of photocatalytic CO_2_ reduction.

**Table S2.** Summary of C_2_H_6_ selectivity and the relevant reaction conditions of photocatalytic CO_2_ reduction systems reported in recent years.

**IV.** **References**

**I. Supplementary Experimental Details**

**Materials:** Bismuth nitrate pentahydrate (Bi(NO_3_)_3_·5H_2_O), chloroplatinic acid hexahydrate (H_2_PtCl_6_·6H_2_O) and palladium dichloride (PdCl_2_) were purchased from Shanghai Aladdin Reagent Co., LTD. Sodium chloride (NaCl), potassium bromide (KBr), potassium iodide (KI), mannitol (C_6_H_14_O_6_), urea (CON_2_H_4_), sodium borohydride (NaBH_4_) were obtained from Shanghai Sinopharm Chemical Reagent Co., Ltd.. Chloroauric acid trihydrate (HAuCl_4_.3H_2_O) was purchased from ACROS ORGANICS. Commercial TiO_2_ (P25) was supplied from Evonik-Degussa. No additional treatment was applied before using these chemicals for synthesis.

**Synthesis of BiOCl and g-C_3_N_4_**: In a typical procedure, 0.9701 g of Bi(NO_3_)_3_·5H_2_O was dissolved into 50 mL of mannitol solution (0.1 mol L^-1^). Then, 0.1168 g of NaCl was added into the solution. After the mixed solution was stirred for 4 h at room temperature, the solid product was collected by centrifugation and washed by deionized water *via* ultrasonic treatment and centrifugation for six times. The BiOCl sample was finally obtained by [freeze](javascript:;) [drying](javascript:;). BiOCl-1 was synthesized under identical conditions as the BiOCl sample, except that deionized water is used instead of mannitol solution (0.1 mol L^-1^). In the typical synthesis of a g-C_3_N_4_ **s**ample, 5 g of urea was put into a covered crucible, heated to 500 °C at a ramp rate of 2 °C min^-1^ in a tube furnace under air condition, and then maintained at this temperature for an additional 2 h.^[1]^ After being cooled down to room temperature, the resultant powders were collected.

**Synthesis of metal-supported (M=Pt, Pd, and Au) semiconductors:** Typically, 0.1 g of BiOCl sample and 0.133 mL of H_2_PtCl_6_·6H_2_O solution (the content of Pt is 7.5 g L^-1^) were dispersed in 10 mL of deionized water with sonication for 30 min. Then, 2 mL of fresh NaBH_4_ solution (0.02 mol L^-1^) was added dropwise into the mixed solution under stirring in an ice–water bath. After stirring for 10 min, the solid product was collected and washed with distilled water for six times. The 1 wt% Pt supported BiOCl sample (1% Pt/BiOCl) was ﬁnally obtained by drying under vacuum at 60 °C for 12 h. 1% Pd/BiOCl and 1% Au/BiOCl samples were prepared with the same procedure, but by replacing the H_2_PtCl_6_·6H_2_O solution with 1 mL of PdCl_2_ solution (the content of Pd is 1 g L^-1^) and 0.2 mL of HAuCl_4_·3H_2_O solution (the content of Au is 5 g L^-1^). 0.5 wt% and 1.5 wt% metal (M=Pt, Pd, and Au) supported BiOCl samples were prepared by changing the amount of metal precursors added. The synthesis of 1% Pt, Pd and Au supported other semiconductors (P25, g-C_3_N_4_ and BiOCl-1) was similar to the method of synthesizing metal-supported (Pt, Pd, and Au) BiOCl samples, except for changing the corresponding metal precursors or photocatalysts.

**Characterizations:** The X-ray diffraction (XRD) patterns were recorded on a diffractometer (D8, Bruker Co., Germany) with Cu Kα radiation (λ=1.5406 Å). The morphology and energy dispersive X-ray spectroscopy (EDX) of samples were characterized by a scanning electron microscope (ZEISS, GeminiSEM 300). TEM images were performed on JEOL JSM-2100. X–ray photoelectron spectroscopy (XPS) data were detected with a VG Multilab 2000 photoelectron spectrometer (Al Kα, 2×10^−6^ Pa). The Brunauer–Emmett–Teller (BET) specific surface area was analyzed by nitrogen adsorption on a Micromeritics ASAP 2020 nitrogen adsorption apparatus (USA). UV–vis diffuse reflectance spectra (DRS) were recorded on UV–vis spectrophotometers (Hitachi UH 4150) by using BaSO_4_ as a reference. Photoluminescence spectra (PL) were detected with a Shimadzu RF-5301PC fluorescence spectrophotometer from 200 to 800 nm with an excitation wavelength of 270 nm.

CO Temperature-programmed desorption (CO-TPD) measurements were conducted on a Chemisorption Analyzer (Chemstar TPx). First, the sample was treated at 100 ^o^C in an Ar atmosphere for 1 h to remove the adsorbed substances on the sample surface. Then, it was cooled to 30 ^o^C to absorb CO for 1 h, after which, the sample was blown in an He atmosphere for 30 minutes to remove the physical adsorbed CO molecules on the surface. Subsequently, CO molecules adsorbed on the sample were desorbed at a rate of 10 ^o^C min^-1^ up to 800 ^o^C. The desorbed CO were detected by a thermal conductivity detector (TCD), and mass spectrometry (MS) was employed to detect the content changes of CO (m/z values of 28).

The electrochemical measurement was detected on a CHI 660E electrochemical system (Shanghai, China) by using a standard three-electrode cell. Photocurrent responses of the photocatalysts were measured at open-circuit potential in 0.5 M Na_2_SO_4_ electrolyte solution upon simulated light irradiation (50 W Xe lamp) light on and off. A platinum wire was taken as the counter electrode and a saturated calomel electrode (SCE) was taken as the reference electrode. The working electrode was prepared according to the following process: 5 mg of as-prepared sample was mixed with 0.5 mL of deionized water and 20 μL of nafion solution (5%, DuPont) to form a homogeneous ink. Then, 40 μL of the catalyst ink was deposited on a 1×1 cm^2^ indium-tin oxide (ITO) glass electrode. The as-prepared electrode was then dried at 60 °C for 2 h in an electric oven. Electrochemical impedance spectroscopy (EIS) was carried out at open-circuit potential in 0.5 M KCl solution (containing 5.0 mM K_3_[Fe(CN)_6_]/K_4_[Fe(CN)_6_]). The working electrode was prepared according to the following process: 10 μL of the catalyst ink was deposited on the center of a glassy carbon electrode and dried in an oven at 40 ^o^C.

In situ diffuse reflectance infrared Fourier transform spectroscopy (DRIFTS) of CO_2_ adsorption and photoreduction was recorded on a Nicolet iS50 (Thermo) infrared spectrometer equipped with a mercury cadmium telluride (MCT) detector. Typically, the sample was pressed into the sample cell, which was then thoroughly vacuum-treated to completely remove air. Then, the sample cell was heated to 100 ^o^C and maintained at this temperature for an additional 30 min to remove some possible residue. After the sample cell cooled down naturally to room temperature, the background was collected. After that, CO_2_ and H_2_O vapor were pushed into the sample cell by an injection syringe. Prior to light illumination, the sample cell was kept in the dark for 30 min to establish an adsorption-desorption equilibrium of CO_2_ and aqueous vapor over the photocatalysts. Then, a 300 W Xe lamp was employed as the light source. Meanwhile, the IR spectrum was recorded at certain time intervals.

**Photocatalytic CO_2_ reduction measurement:** The photocatalytic CO_2_ reduction experiment was carried out in a Labsolar-6A closed gas system (Beijing Perfectlight Technology Co., Ltd.). In a typical photocatalytic reaction, 20 mg of as-prepared sample with 1 mL of ultra-pure water was well-dispersed on a [glass](javascript:;) [culture](javascript:;) [dish](javascript:;) with a diameter of 6 cm, which was then placed at the bottom of a glass reactor. The reaction setup was sealed with a quartz cover. After thorough vacuum treatment of the system, high-purity CO_2_ (99.9%) was used to wash the system for five times, and then high-purity CO_2_ was finally filled into the glass reactor to achieve a pressure of 0.07 MPa. Prior to light illumination, the glass reactor was kept in the dark for 30 min to establish an adsorption–desorption equilibrium of CO_2_ and aqueous vapor over the photocatalysts. Then, a 300 W Xe lamp (PLS–SXE300D, Beijing Perfectlight Technology Co., Ltd.) was employed as the light source. The reaction temperature was maintained at 10 °C by a circulating water bath. A full-automatic on-line gas chromatograph (GC9790Plus, FuLi instruments) was employed to determine the gas product at fixed intervals. Argon (Ar) gas was used as the carrier gas. H_2_ and O_2_ were detected by a TCD. CO was detected by an FID after bypassing a methane converter. CH_4_, C_2_H_4_ and C_2_H_6_ were detected by the FID. In the control experiment, high-purity CO_2_ (99.9%) was replaced by high-purity Ar (99.9%) under identical reaction conditions. The control experiment was also performed under dark conditions without changing other reaction conditions. ^13^CO_2_ isotope labeling experiments were conducted on the 1% Pd/BiOCl sample under identical conditions by using ^13^CO_2_ (Wuhan Isotope Technology Co.,Ltd). The resulting gas products were then analyzed by gas chromatography-mass spectrometry (GC-MS, Agilent 5977B). Product selectivity is evaluated based on the required electrons using the following equation：

$$\text{Selectivity=}\frac{\text{n(Product)*number of electrons}}{\text{2n(CO)+8n(}\text{CH}_{\text{4}}\text{)+12n(}\text{C}_{\text{2}}\text{H}_{\text{4}}\text{)+14n(}\text{C}_{\text{2}}\text{H}_{\text{6}}\text{)+2n(}\text{H}_{\text{2}}\text{)}}\text{ }\text{Equation (}\text{S}\text{1)}$$

where n(CO), n(CH_4_), n(C_2_H_4_), n(C_2_H_6_), and n(H_2_) are the number of moles of CO, CH_4_, C_2_H_4_, C_2_H_6_, and H_2_ formed at a given time, respectively.

**The calculation of solar-to-CO/CH_4_/C_2_H_4_/C_2_H_6_ conversion efﬁciency (η):** In accordance with literature,^[2]^ the solar-to- CO/CH_4_/C_2_H_4_/C_2_H_6_ conversion efﬁciency (η) was calculated as follows:

$$\text{η}\text{=}\frac{\begin{aligned} \text{[R(CO)}\text{×}\text{Δ}\text{G}^{\text{0}}\text{(CO)+R(}\text{CH}_{\text{4}}\text{)}\text{×}\text{Δ}\text{G}^{\text{0}}\text{(}\text{CH}_{\text{4}}\text{)+R(}\text{C}_{\text{2}}\text{H}_{\text{4}}\text{)}\text{×}\text{Δ}\text{G}^{\text{0}}\text{(}\text{C}_{\text{2}}\text{H}_{\text{4}}\text{)} \\ \text{+R(}\text{C}_{\text{2}}\text{H}_{\text{6}}\text{)}\text{×}\text{Δ}\text{G}^{\text{0}}\text{(}\text{C}_{\text{2}}\text{H}_{\text{6}}\text{)]} \end{aligned}}{\text{[P}\text{×}\text{S}\text{]}}\text{×100\%}\text{ }\text{ }\text{Equation (}\text{S}\text{2}\text{)}$$

where R(CO), R(CH_4_), R(C_2_H_4_), R(C_2_H_6_), ΔG^O^(CO), ΔG^O^(CH_4_), ΔG^O^(C_2_H_4_), ΔG^O^(C_2_H_6_), P, and S denote the rate of CO, CH_4_, C_2_H_4_, and C_2_H_6_ evolution (mol s^−1^) in the CO_2_ photoreduction system, the change in the Gibbs free energy that accompanies the reduction of CO_2_ to CO (257 × 10^3^ J mol^-1^), CH_4_ (818 × 10^3^ J mol^-1^), C_2_H_4_ (1331 × 10^3^ J mol^-1^), and C_2_H_6_ (1467 × 10^3^ J mol^-1^), the solar light energy intensity (0.13 Wcm^-2^), and the illumination area (28.26 cm^2^), respectively.

**Computational Methods:** We carried out all DFT calculations using the Vienna *ab initio* simulation (VASP5.4.4) code.^[3]^ The exchange-correlation is simulated with the PBE functional and ion-electron interactions were described by the PAW method.^[4]^ The vdWs interaction was included by using the empirical DFT-D3 method.^[5]^ The Monkhorst-Pack-grid-mesh-based Brillouin zone *k*-points are set as 2×2×1 for all periodic structures with a cutoff energy of 450 eV. The convergence criteria are set as 0.01 eV A^-1^ and 10^‑5^ eV in force and energy, respectively. A 20 Å vacuum layer along the *z* direction is employed to avoid interlayer interference. The adsorption energy (∆*E*adsorption) is calculated as

∆*E*adsorption=*E*_total_ –*E*_substrate_ – *E*_adsorbate_ ,

where *E*_substrate_ is the energy of the catalyst surface, *E*_adsorbate_ represents the energy of the intermediate, and *E*_total_ represents the total energy of the single molecule adsorption system. The free energy calculation of species adsorption ($\Delta G$) is based on Nørskov *et al*’s hydrogen electrode model,^[6]^ as follows:

$\Delta G\text{= }\Delta E+\Delta E_{\text{ZPE}}-T\Delta S$ (Equation S3)

Herein, Δ*E*, Δ*E*_ZPE_, and Δ*S* respectively represent the changes in electronic energy, zero‑point energy, and entropy caused by the adsorption of the intermediate. The entropy of the H^+^+e^-^ pair is approximated to be half of that of H_2_ in standard conditions.^[7]^

**II. Supplementary Figures**


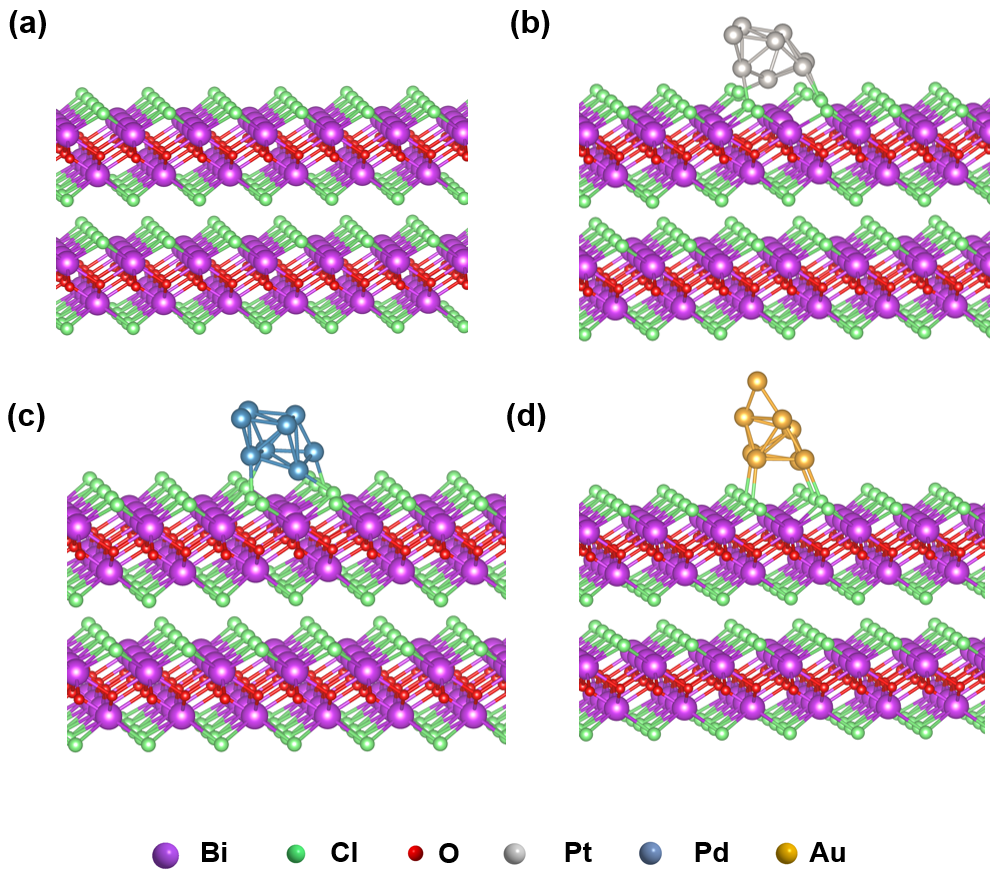


**Figure S1.** Computational models of BiOCl-(001) (a), Pt/BiOCl-(001) (b), Pd/BiOCl-(001) (c), and Au/BiOCl-(001) (d).


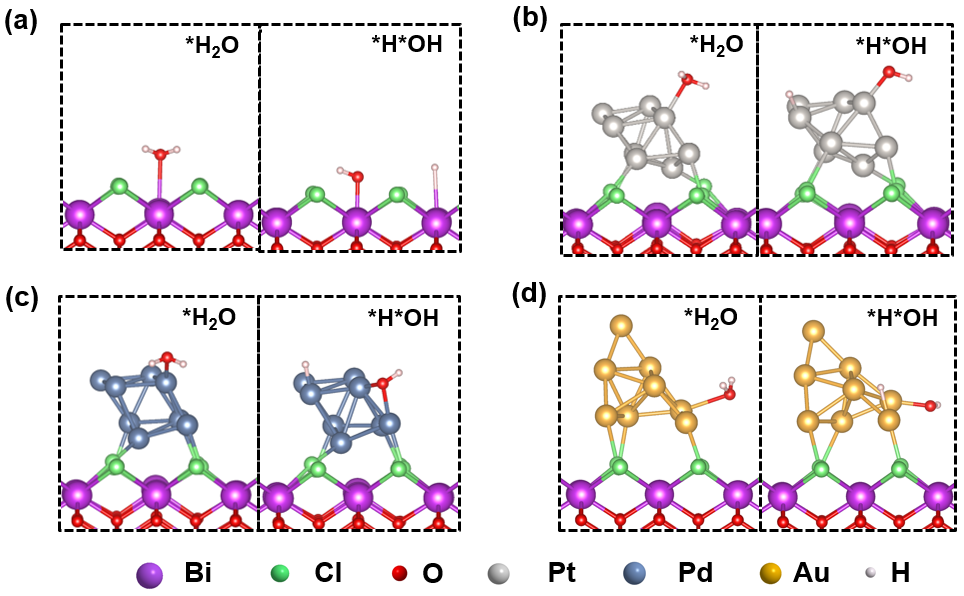


**Figure S2.** Adsorption configurations of various intermediates involved in H_2_O dissociation on BiOCl (a), Pt/BiOCl (b), Pd/BiOCl (c), and Au/BiOCl (d).


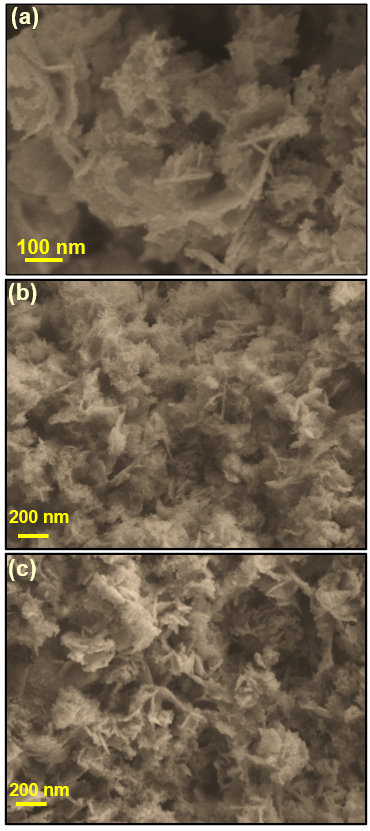


**Figure S3.** SEM images of BiOCl (a), 1% Pt/BiOCl (b), and 1% Pd/BiOCl (c) samples.


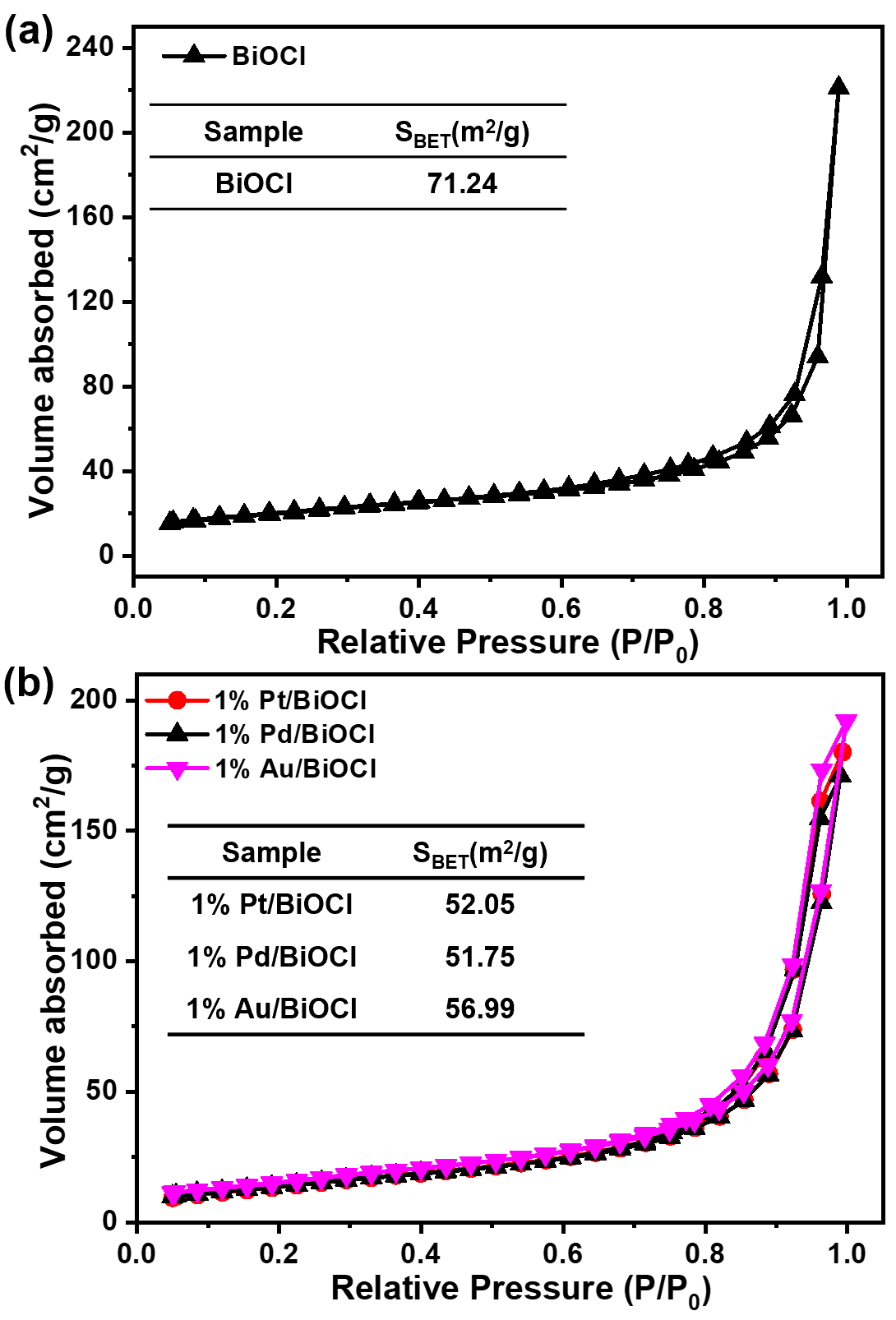


**Figure S4.** N_2_ adsorption–desorption isotherm and BET speciﬁc surface areas of BiOCl (a), and 1% Pt/BiOCl, 1% Pd/BiOCl, and 1% Au/BiOCl (b) samples.


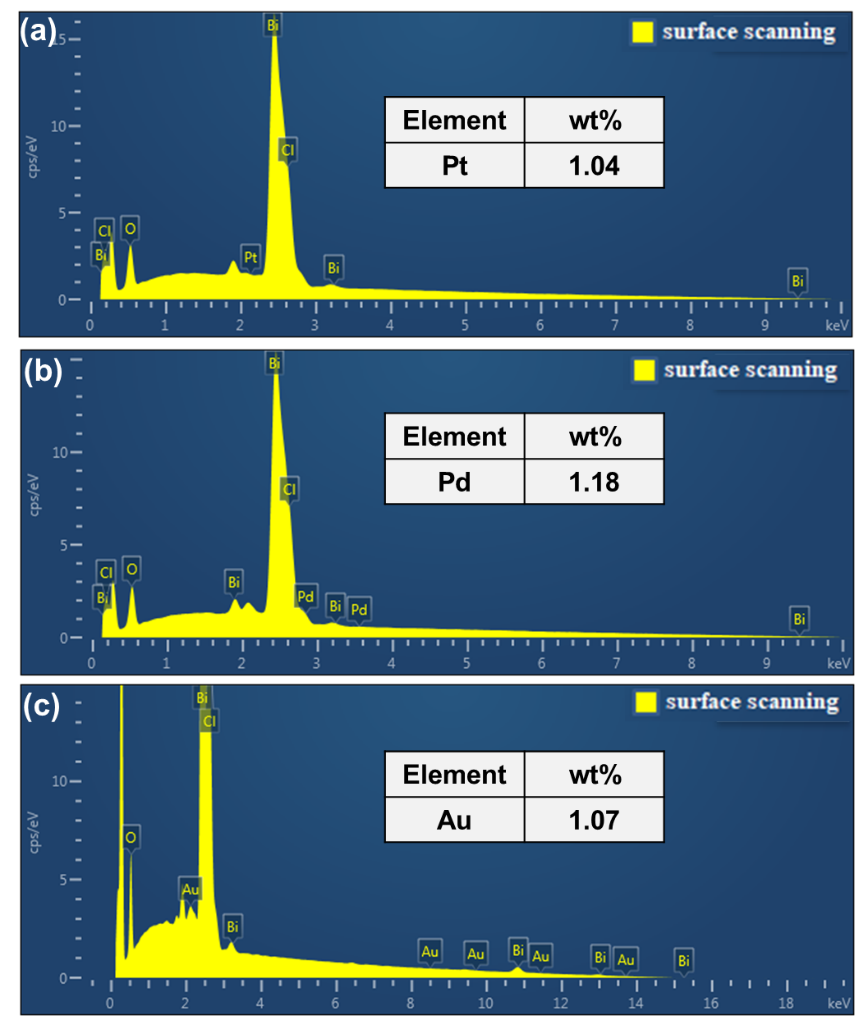


**Figure S5.** Energy-dispersive X-ray spectroscopy of 1% Pt/BiOCl (a), 1% Pd/BiOCl (b), and 1% Au/BiOCl (c) samples.


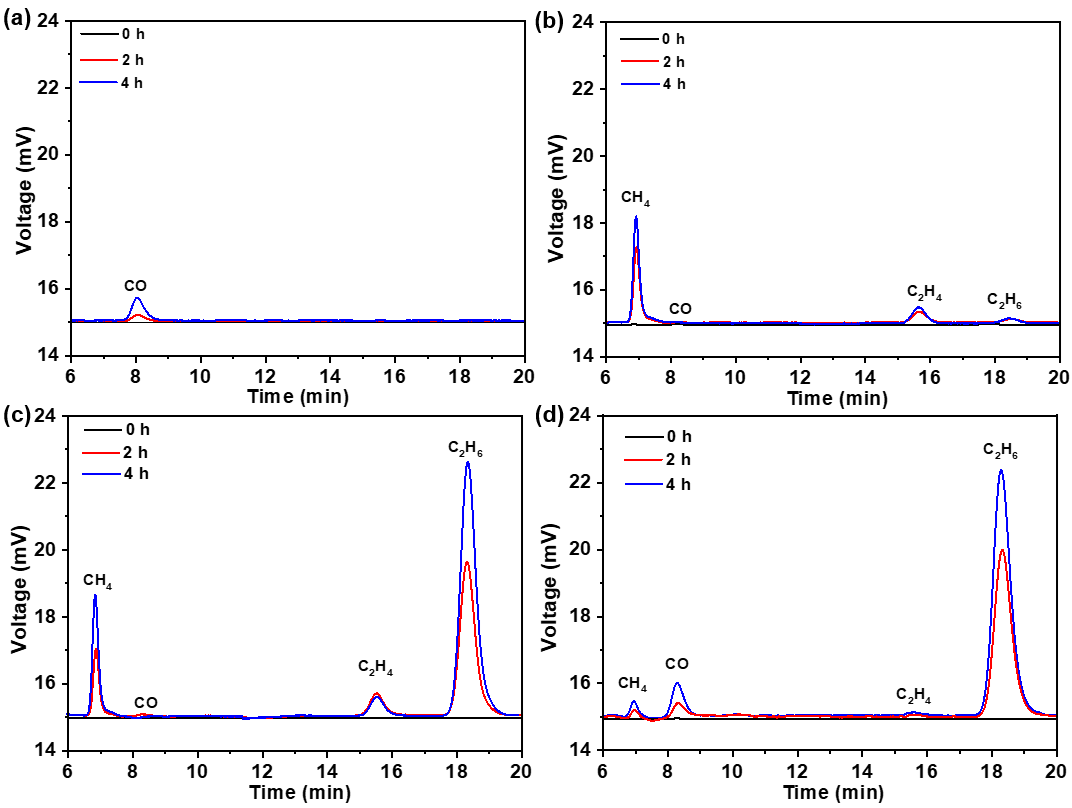


**Figure S6.** Gas chromatograms of the products generated over BiOCl (a), 1% Pt/BiOCl (b), 1% Pd/BiOCl (c), and 1% Au/BiOCl (d) samples during photocatalytic CO_2_ reduction after 0, 2 and 4 h.


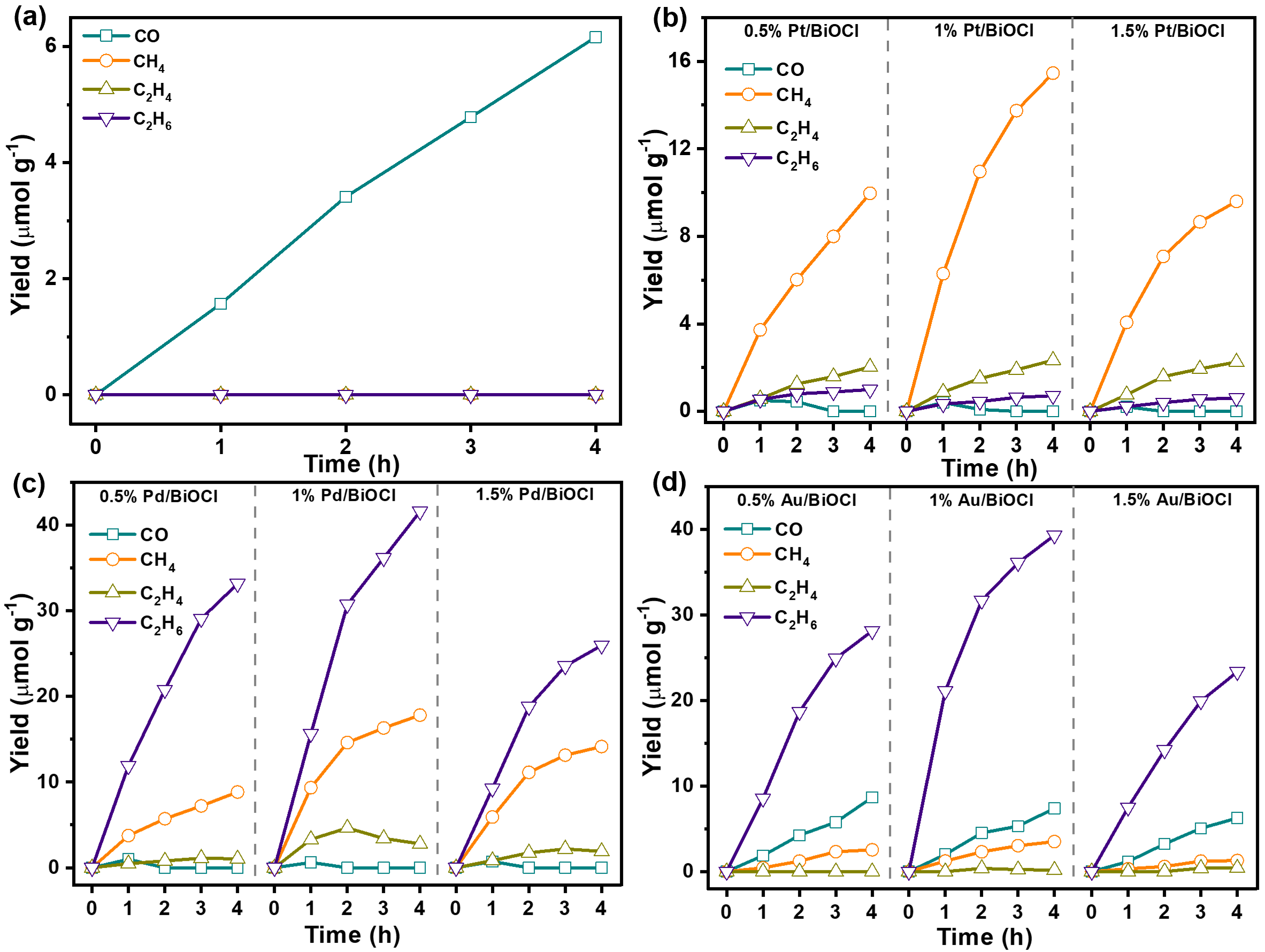


**Figure S7.** The yield of different products versus irradiation time for the photocatalytic CO_2_ reduction reaction over BiOCl (a), and 0.5%, 1%, and 1.5% Pt (b), 0.5%, 1%, and 1.5% Pd (c), and 0.5%, 1%, and 1.5% Au (d) supported BiOCl samples.


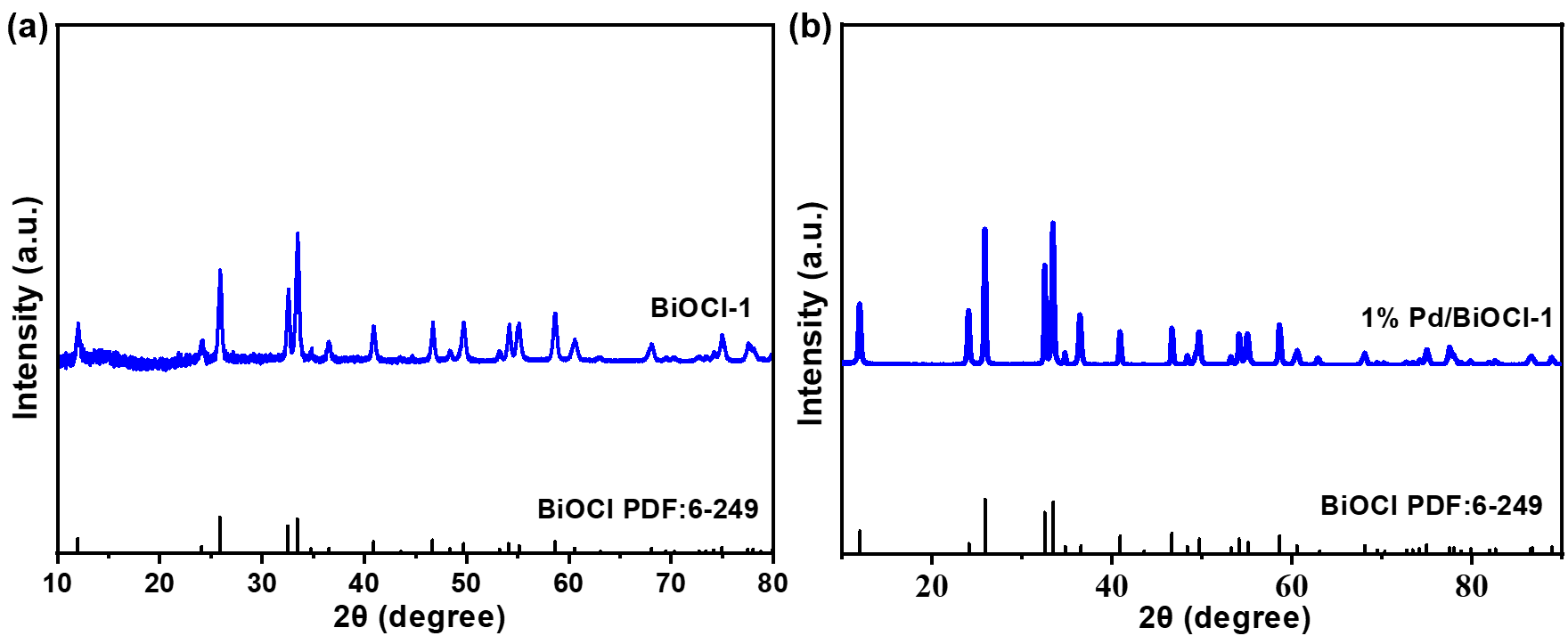


**Figure S8.** XRD patterns of the BiOCl-1 (a) and 1% Pd/BiOCl-1 (b) samples.

The powder XRD pattern of the BiOCl-1 sample is shown in Figure S8(a). All characteristic diffraction peaks of the BiOCl-1 sample were well-indexed to the standard XRD pattern of BiOCl (PDF 6-249). No other peak was observed, indicative of the high purity of the prepared BiOCl-1 sample. As shown in Figure S8 (b), the characteristic diffraction peaks of the 1% Pd/BiOCl-1 sample were also well-indexed to the standard XRD pattern of BiOCl (PDF 6-249), demonstrating that metal loading did not affect the crystallinity and ﬁne structure of BiOCl-1.


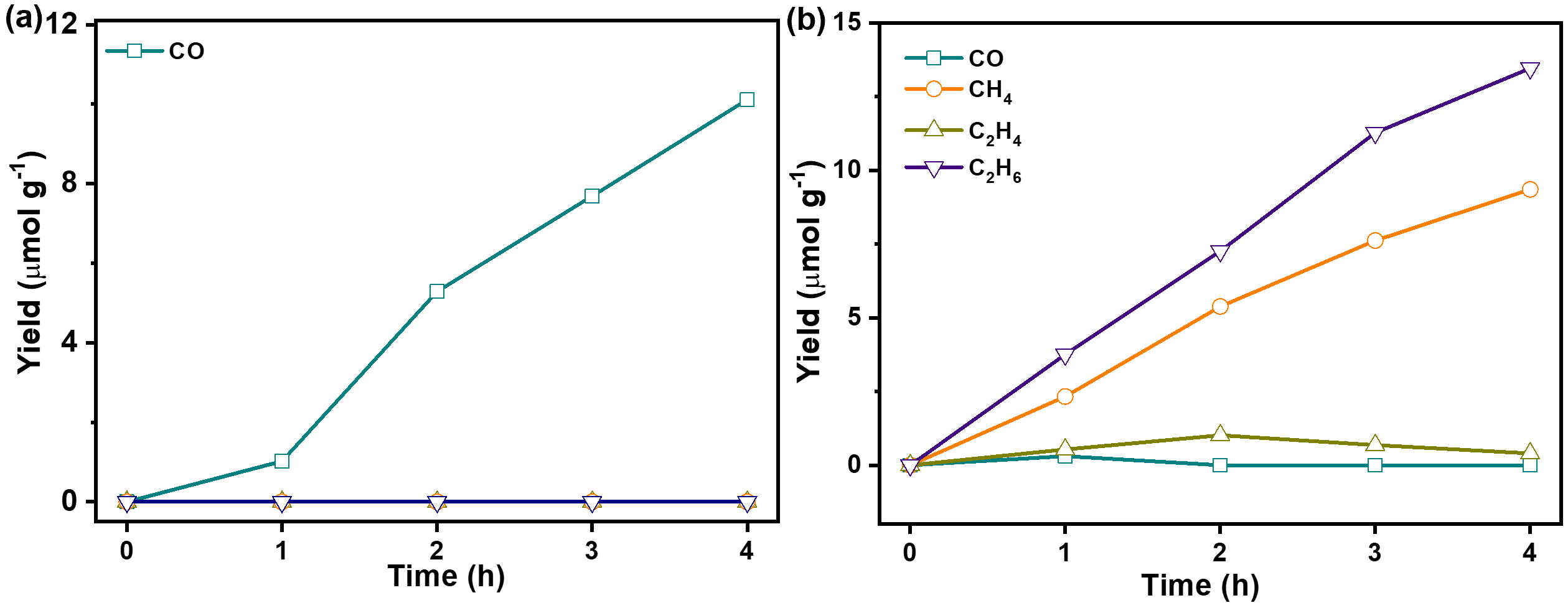


**Figure S9.** The yield of different products versus irradiation time for the photocatalytic CO_2_ reduction reaction over BiOCl-1 (a) and 1% Pd/BiOCl-1 (b) samples.

As displayed in Figure S9(a), the CO yield gradually improved with illumination time, and the total yield of CO achieved during 4 h of reaction was 10.11 μmol g^-1^ over BiOCl-1 samples. The products CH_4_, C_2_H_6_, and C_2_H_4_ were also detected in the photocatalytic CO_2_ reduction reaction system involving the 1% Pd supported BiOCl-1 sample (Figure S9(b)).


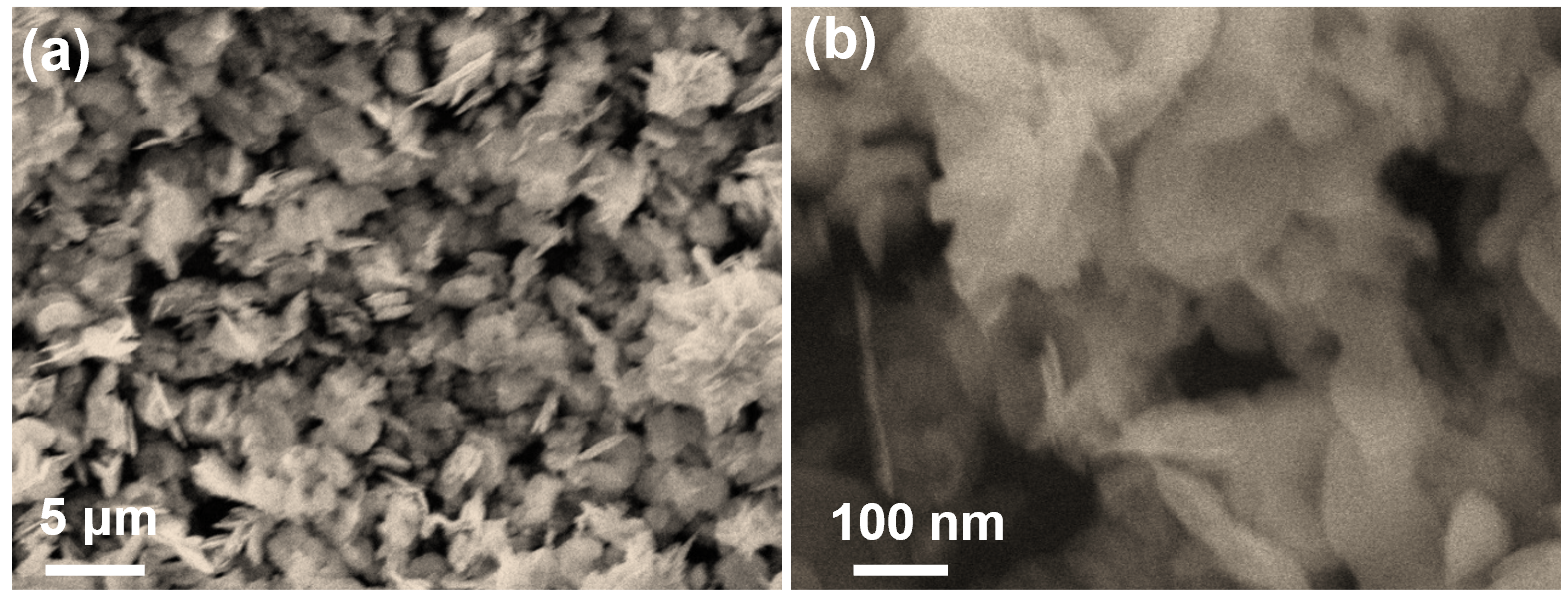


**Figure S10.** SEM images of the BiOCl-1 sample (a) and (b).

The morphology of the as-synthesized BiOCl-1 sample was characterized by SEM. As shown in Figure S10, the prepared BiOCl-1 sample displayed a sheet morphology.


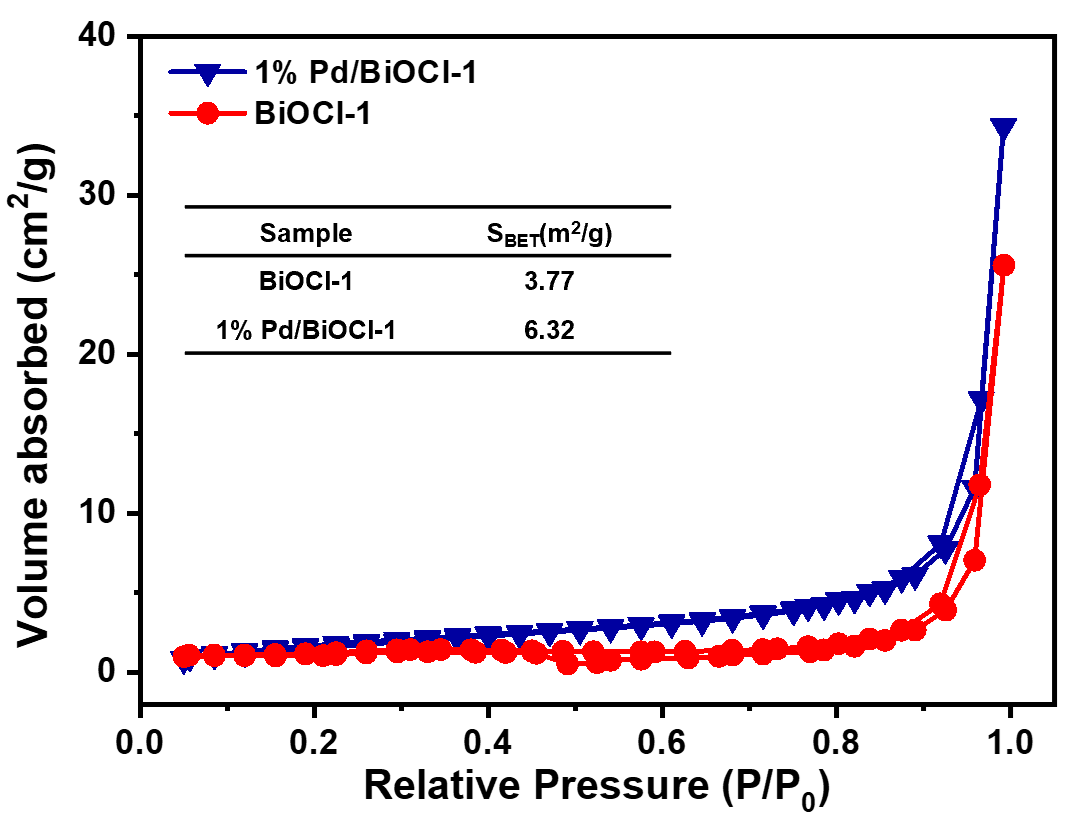


**Figure S11.** N_2_ adsorption–desorption isotherms and BET speciﬁc surface areas of BiOCl-1 and 1% Pd/BiOCl-1 samples.

As displayed in Figure S11, the measured BET speciﬁc surface areas of BiOCl-1 and 1% Pd/BiOCl-1 was 3.77 and 6.32 m^2^ g^-1^, respectively.


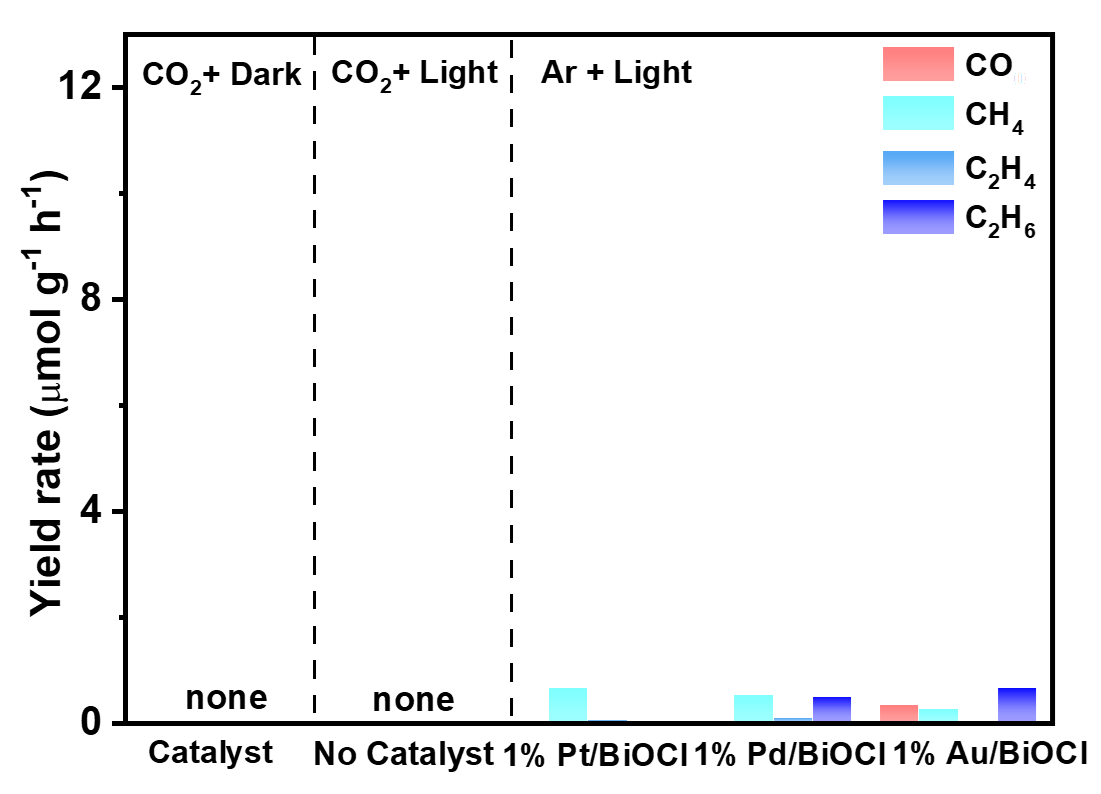


**Figure S12.** PCRR control experiments of 1% M/BiOCl (M=Pt, Pd, and Au) samples.


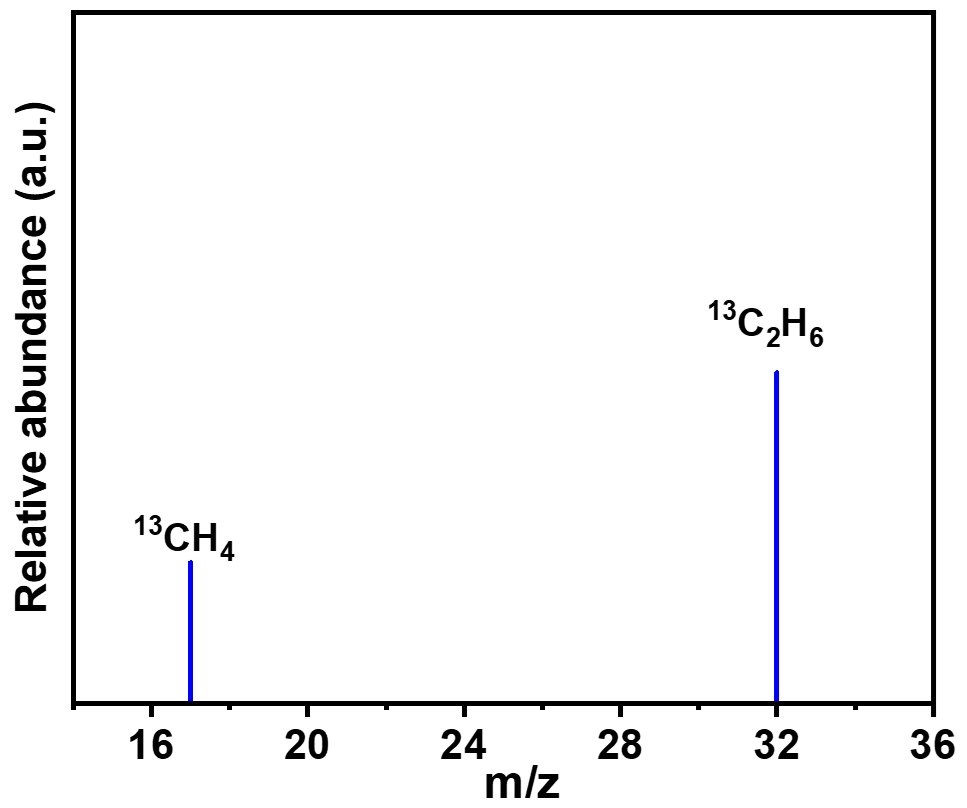


**Figure S13.** Isotope-labeled ^13^CO_2_ experiment of 1% Pd/BiOCl.


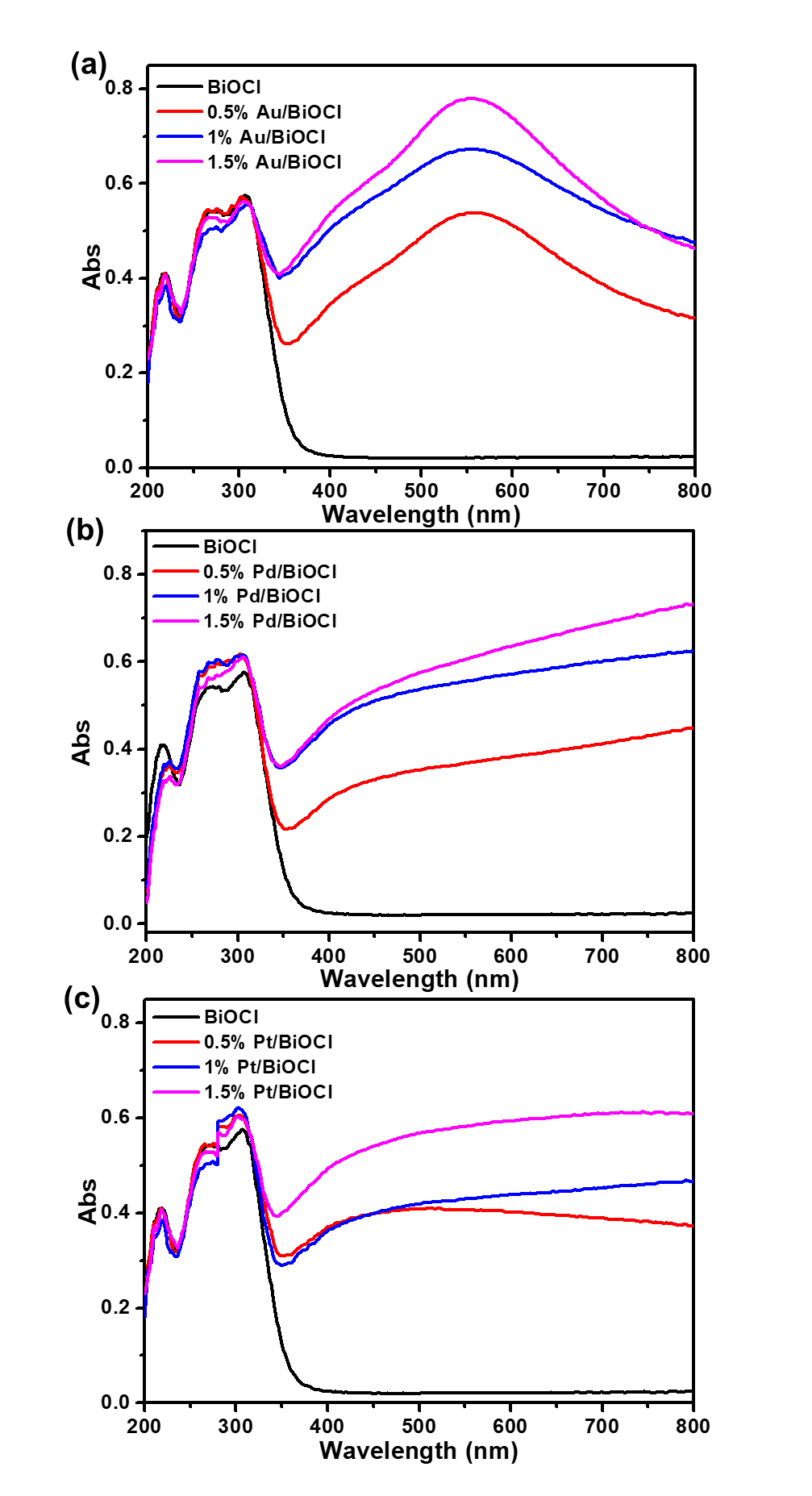


**Figure S14.** The UV-vis diffuse reflectance spectra (DRS) of 0.5%, 1%, and 1.5% Au/BiOCl (a), 0.5%, 1%, and 1.5% Pd/BiOCl (b), and 0.5%, 1%, and 1.5% Pt/BiOCl (c).


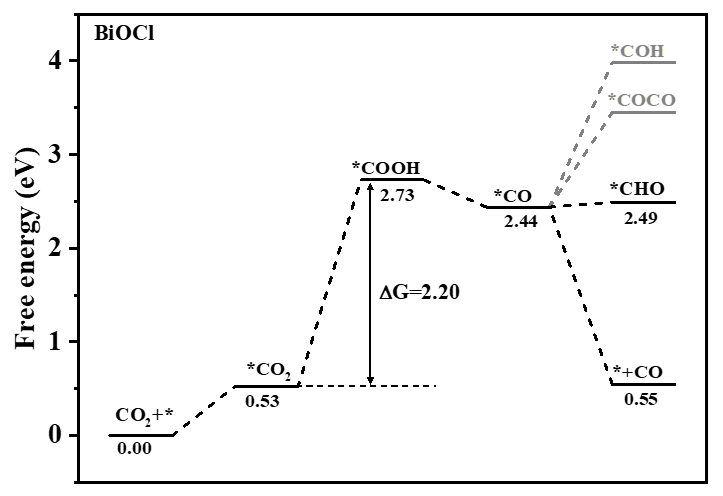


**Figure S15.** Calculated free energy of CO_2_ reduction on BiOCl for the photoreduction of CO_2_ to CO with H_2_O.


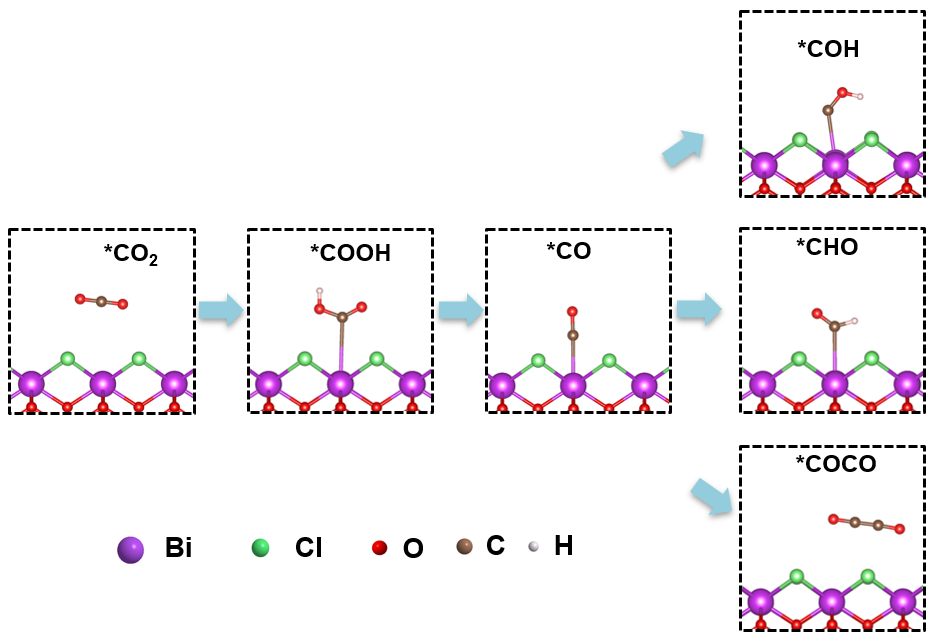


**Figure S16.** Adsorption configurations of the various intermediates involved in photocatalytic CO_2_ reduction over the BiOCl sample.


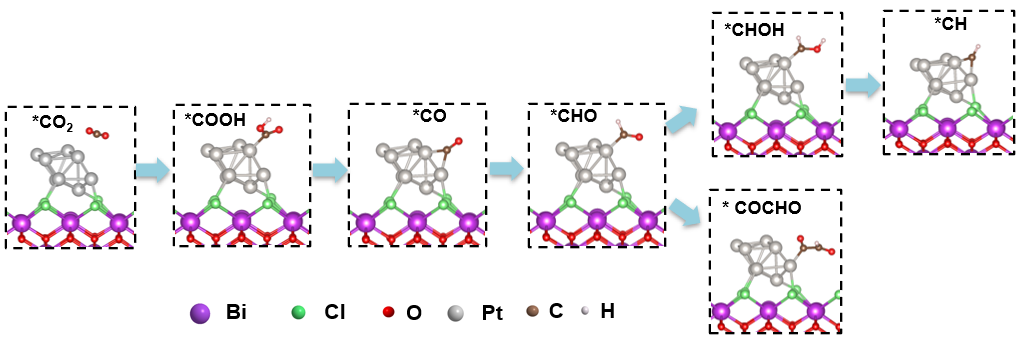


**Figure S17.** Adsorption configurations of the various intermediates involved in photocatalytic CO_2_ reduction over the Pt/BiOCl sample.


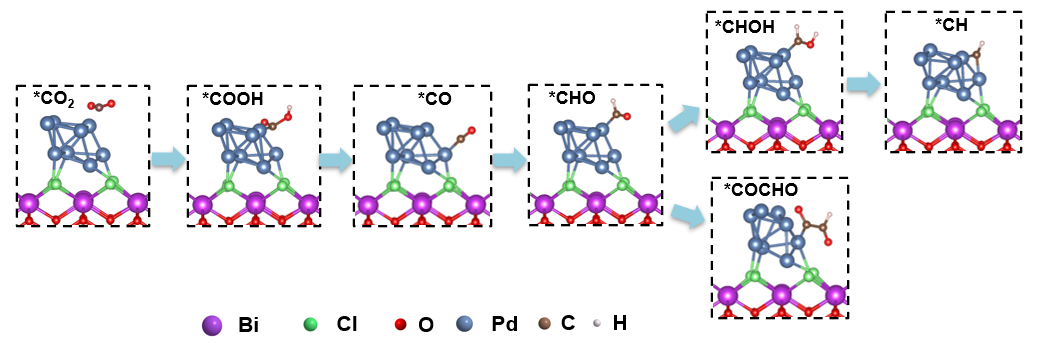


**Figure S18.** Adsorption configurations of the various intermediates involved in photocatalytic CO_2_ reduction over the Pd/BiOCl sample.


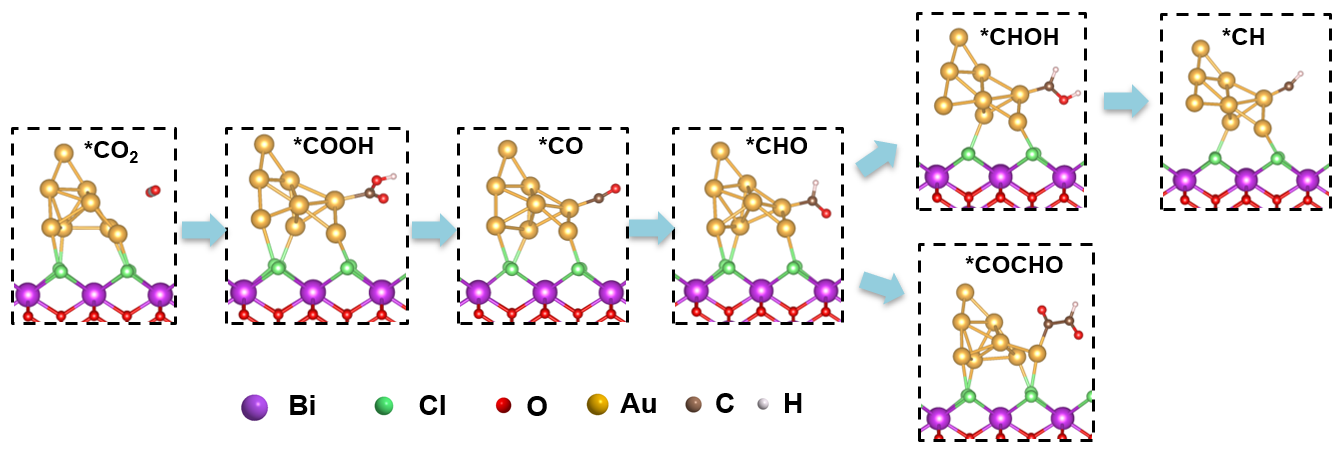


**Figure S19.** Adsorption configurations of the various intermediates involved in photocatalytic CO_2_ reduction over the Au/BiOCl sample.


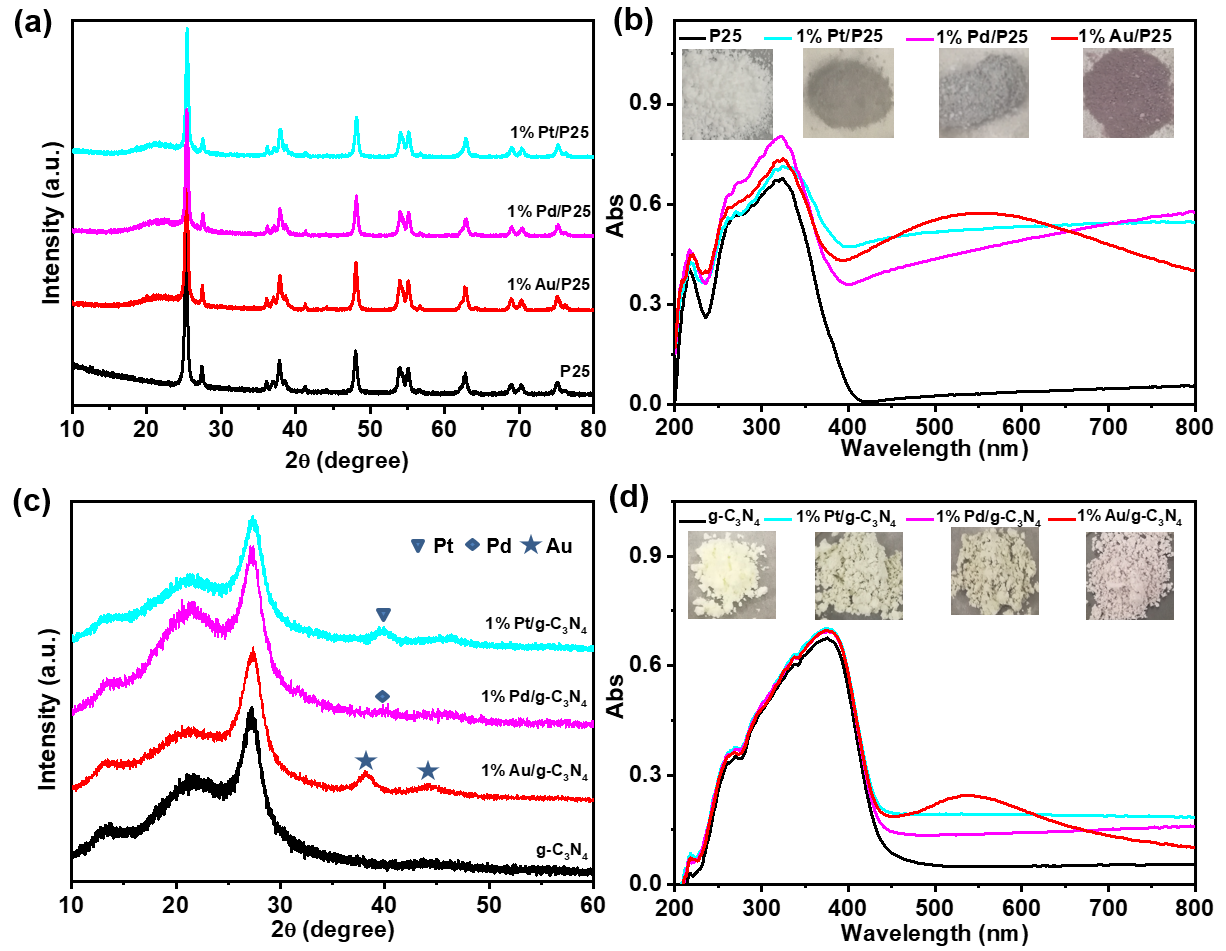


**Figure S20.** XRD (a and c) and DRS (b and d) of metal-supported (1% Pt, 1% Pd, and 1% Au) P25 (a and b) and metal-supported (1% Pt, 1% Pd, and 1% Au) g-C_3_N_4_ (c and d) samples.


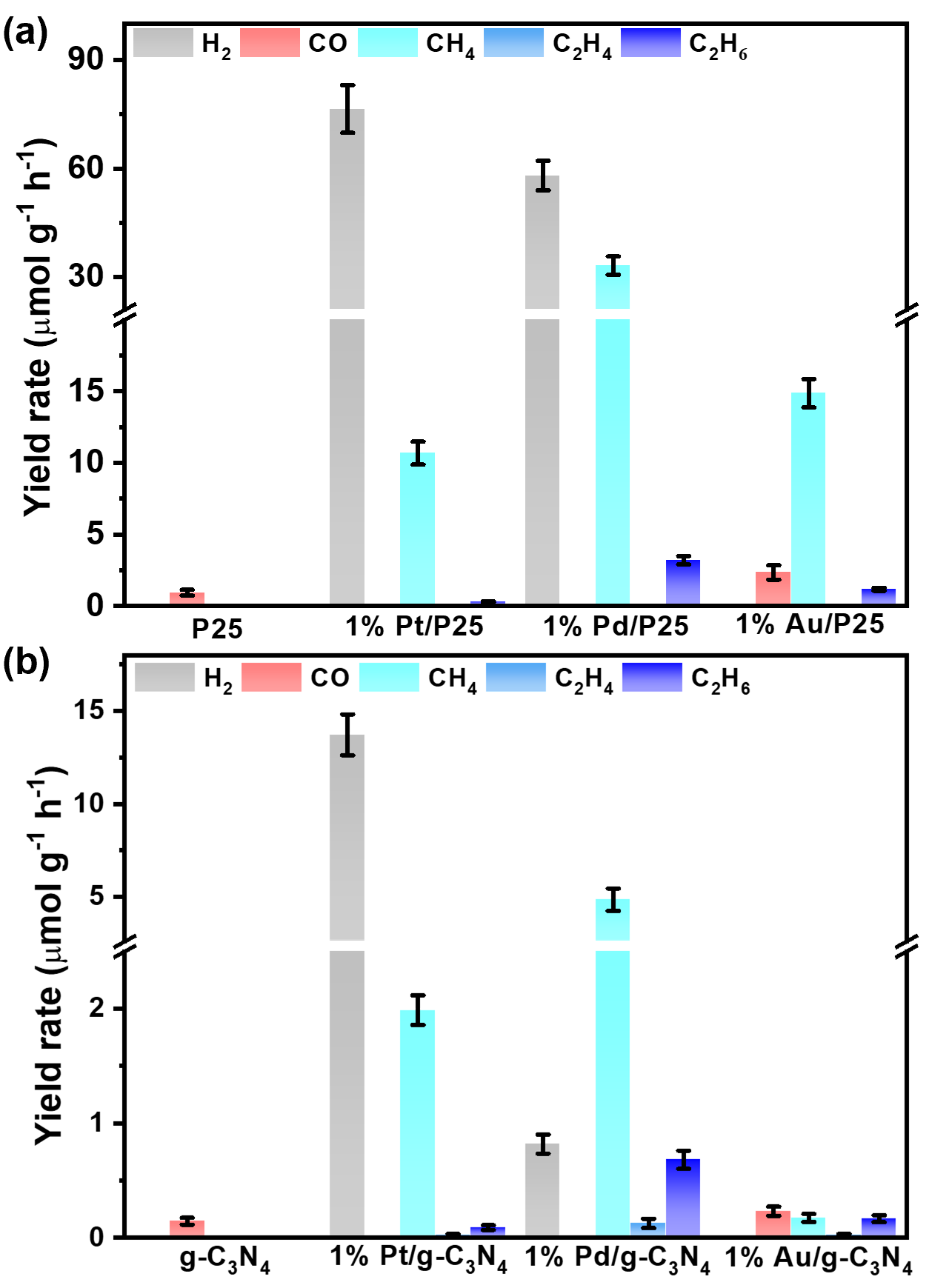


**Figure S21.** The yield rate of P25 and metal-supported (1% Pt, 1% Pd, and 1% Au) P25 (a), and g-C_3_N_4_ and metal-supported (1% Pt, 1% Pd, and 1% Au) g-C_3_N_4_ (b) samples after 4 h of photocatalytic CO_2_ reduction.

**III. Supplementary Tables**

**Table S1.** The yield and selectivity (%) of different products over 1% Pt, 1% Pd, and 1% Au supported BiOCl, P25, and g-C_3_N_4_ samples after 4 h of photocatalytic CO_2_ reduction.

| Catalyst | Yield (μmol g^-1^) | | | | |  | Selectivity (%)*^a^* | | | | |
| --- | --- | --- | --- | --- | --- | --- | --- | --- | --- | --- | --- |
|  | CO | CH_4_ | C_2_H_4_ | C_2_H_6_ | H_2_ |  | CO | CH_4_ | C_2_H_4_ | C_2_H_6_ | H_2_ |
| BiOCl | 6.16 | nd*^b^* | nd*^b^* | nd*^b^* | nd*^b^* |  | 100 | 0 | 0 | 0 | 0 |
| 1% Pt/BiOCl | nd*^b^* | 15.46 | 2.33 | 0.71 | nd*^b^* |  | 0 | 76.51 | 17.33 | 6.16 | 0 |
| 1% Pd/BiOCl | nd*^b^* | 17.80 | 2.78 | 41.61 | nd*^b^* |  | 0 | 18.79 | 4.40 | 76.81 | 0 |
| 1% Au/BiOCl | 7.40 | 3.50 | 0.17 | 39.31 | nd*^b^* |  | 2.49 | 4.70 | 0.35 | 92.46 | 0 |
| P25 | 3.71 | nd*^b^* | nd*^b^* | nd*^b^* | nd*^b^* |  | 100 | 0 | 0 | 0 | 0 |
| 1% Pt/P25 | nd*^b^* | 42.71 | nd*^b^* | 1.17 | 305.68 |  | 0 | 35.24 | 0 | 1.69 | 63.06 |
| 1% Pd/P25 | nd*^b^* | 132.73 | nd*^b^* | 12.79 | 232.06 |  | 0 | 62.28 | 0 | 10.50 | 27.22 |
| 1% Au/P25 | 9.39 | 59.48 | 0.60 | 4.68 | nd*^b^* |  | 3.31 | 83.87 | 1.28 | 11.54 | 0 |
| g-C_3_N_4_ | 0.58 | nd*^b^* | nd*^b^* | nd*^b^* | nd*^b^* |  | 100 | 0 | 0 | 0 | 0 |
| 1% Pt/g-C_3_N_4_ | nd*^b^* | 6.84 | 0.09 | 0.24 | 54.89 |  | 0 | 32.37 | 0.66 | 1.98 | 64.98 |
| 1% Pd/g-C_3_N_4_ | nd*^b^* | 18.76 | 0.50 | 2.69 | 3.27 |  | 0 | 74.96 | 2.98 | 18.79 | 3.27 |
| 1% Au/g-C_3_N_4_ | 1.19 | 0.69 | 0.09 | 0.66 | nd*^b^* |  | 13.04 | 30.26 | 6.05 | 50.65 | 0 |

*^a^*The reaction selectivity was calculated by Equation S1.*^b^*nd denotes not detected.

**Table S2.** Summary of C_2_H_6_ selectivity and the relevant reaction conditions of photocatalytic CO_2_ reduction systems reported in recent years.^[8]^

| Photocatalyst | Products | C_2_H_6_  selectivity | Reaction conditions | | | Ref. |
| --- | --- | --- | --- | --- | --- | --- |
| 1% Au/BiOCl | CO, CH_4_, C_2_H_4_, C_2_H_6_ | 92.46% | CO_2_, 1mL H_2_O, 300 W Xe lamp | | This work | |
| 1% Pd/BiOCl | CH_4_, C_2_H_4_, C_2_H_6_ | 76.81% | CO_2_, 1mL H_2_O, 300 W Xe lamp | This work | | |
| Au@TiO_2_ | CH_4_, C_2_H_6_ | 53.70% | moist CO_2_, Hg lamp @ 254 nm, 20 mW/cm^2^ | | | ^[9]^ |
| Au NPs | CH_4_, C_2_H_6_ | 40% | moist CO_2_ using isopropanol as sacriﬁcial, (λ > 488 nm, 750 mW/cm^2^) | | | ^[10]^ |
| AuPd/(101) TiO_2_ | CH_4_, C_2_H_4_, C_2_H_6_ | 7.83% | NaHCO_3_ aqueous dispersion CO_2_ saturated, 300 W Xe lamp | | | ^[11]^ |
| PtCu/TiO_2_ nanotubes | CH_4_, C_2_H_4_, C_2_H_6_ | 20.99% | CO_2_ (0.998% in N_2_) ﬂow through H_2_O, 100 mW/cm^2^ | | | ^[12]^ |
| Naﬁon/Pd-TiO_2_ | CH_4_, C_2_H_6_ | 6.40% | NaHCO_3_ aqueous dispersion CO_2_ saturated, 300 W Xe lamp (λ > 300 nm) | | | ^[13]^ |
| Pt- graphene /defect-induced TiO_2_ | C_2_H_4_, C_2_H_6_ | 25.75% | moist CO_2_, sun simulated light (100 mW/cm^2^), reaction performed under continuous ﬂow | | | ^[14]^ |
| Graphene oxide–supported  oxygen–rich TiO_2_ | CO, CH_4_, C_2_H_4_, C_2_H_6_ | 4.91% | CO_2_ ﬂow through H_2_O, AM 1.5 filter | | | ^[15]^ |
| TiO_2_-graphene | CH_4_, C_2_H_6_ | 78.60% | CO_2_, 0.4 mL H_2_O, 300 W Xe lamp | | | ^[16]^ |
| Cu and Co-codoped TiO_2_ | CO, CH_4_, C_2_H_6_, C_3_H_8_ | 71.55% | O_2_, 1mL H_2_O, 300 W Xe lamp | | | ^[17]^ |
| CdS/Cu-Na_x_H_2−x_Ti_3_O_7_ nanotubes | C_2_H_4_, C_2_H_6_, C_3_H_6_, C_3_H_8_ | 31.1% | aqueous dispersion, CO_2_ saturated, 450 W Xe lamp (λ > 420 nm) | | | ^[18]^ |

**IV. References:**

[1] W.-J. Ong, L.-L. Tan, S.-P. Chai, S.-T. Yong, *Chem. Commun.* **2015**, *51*, 858.

[2] a) H. Yu, F. Chen, X. Li, H. Huang, Q. Zhang, S. Su, K. Wang, E. Mao, B. Mei, G. Mul, T. Ma, Y. Zhang, *Nat. Commun.* **2021**, *12*, 4594; b) X. Li, Y. Sun, J. Xu, Y. Shao, J. Wu, X. Xu, Y. Pan, H. Ju, J. Zhu, Y. Xie, *Nat. Energy* **2019**, *4*, 690; c) M. R. Siyuan Fang , Jaya Bharti, Erwin Reisner, Marc Robert , Geoffrey A. Ozin, Yun Hang Hu, *Nat. Rev. Method. Prime.* **2023**, *3*, 61.

[3] G. Kresse, J. Furthmuller, *Phys. Rev. B* **1996**, *54*, 11169.

[4] a) J. P. Perdew, K. Burke, M. Ernzerhof, *Phys. Rev. Lett.* **1996**, *77*, 3865; b) B. Hammer, L. B. Hansen, J. K. Norskov, *Phys. Rev. B* **1999**, *59*, 7413.

[5] S. Grimme, *J. Comput. Chem.* **2006**, *27*, 1787.

[6] E. Skulason, V. Tripkovic, M. E. Bjorketun, S. Gudmundsdottir, G. Karlberg, J. Rossmeisl, T. Bligaard, H. Jonsson, J. K. Norskov, *J. Phys. Chem. C* **2010**, *114*, 18182.

[7] G. Gao, A. P. O'Mullane, A. Du, *ACS Catal.* **2017**, *7*, 494.

[8] J. Albero, Y. Peng, H. García, *ACS Catal.* **2020**, *10*, 5734.

[9] W. G. Tu, Y. Zhou, H. J. Li, P. Li, Z. G. Zou, *Nanoscale* **2015**, *7*, 14232.

[10] S. Yu, A. J. Wilson, J. Heo, P. K. Jain, *Nano Lett.* **2018**, *18*, 2189.

[11] Q. Chen, X. Chen, M. Fang, J. Chen, Y. Li, Z. Xie, Q. Kuang, L. Zheng, *J. Mater. Chem. A* **2019**, *7*, 1334.

[12] X. Zhang, F. Han, B. Shi, S. Farsinezhad, G. P. Dechaine, K. Shankar, *Angew. Chem. Int. Ed.* **2012**, *51*, 12732.

[13] W. Kim, T. Seok, W. Choi, *Energy Environ. Sci.* **2012**, *5*, 6066.

[14] S. Sorcar, J. Thompson, Y. Hwang, Y. H. Park, T. Majima, C. A. Grimes, J. R. Durrant, S.-I. In, *Energy Environ. Sci.* **2018**, *11*, 3183.

[15] L. L. Tan, W. J. Ong, S. P. Chai, A. R. Mohamed, *Chem. Eng. J.* **2017**, *308*, 248.

[16] W. Tu, Y. Zhou, Q. Liu, S. Yan, S. Bao, X. Wang, M. Xiao, Z. Zou, *Adv. Funct. Mater.* **2013**, *23*, 1743.

[17] N. Li, B. Wang, Y. Si, F. Xue, J. Zhou, Y. Lu, M. Liu, *ACS Catal.* **2019**, *9*, 5590.

[18] H. Park, H.-H. Ou, A. J. Colussi, M. R. Hoffmann, *J. Phys. Chem. C* **2015**, *119*, 4658.
